# Chitosan-poly(ethylene glycol)-NaClO_4_ solid polymer electrolytes for activated carbon and flexible laser-induced graphene-based all-solid-state supercapacitors

**DOI:** 10.1039/d6ra07397b

**Published:** 2026-10-15

**Authors:** Jadan Resnik Jaleel UC, Somashekara Bhat, Sudhakar YN, Vipin Cyriac

**Affiliations:** a Manipal Institute of Technology, Manipal Academy of Higher Education Manipal India soma.bhat@manipal.edu; b Symbiosis Institute of Technology (SIT), Pune Campus, Symbiosis International (Deemed University) (SIU) Pune-412115 India

## Abstract

The growing demand for safe, flexible, and sustainable energy storage systems has increased research into all-solid-state supercapacitors. In this work, NaClO_4_-doped chitosan-poly(ethylene glycol) (PEG) solid polymer electrolytes (SPEs) were developed and systematically investigated for use in both rigid and flexible all-solid-state supercapacitors. A series of electrolyte compositions containing 0–50 wt% NaClO_4_ (designated CPN0–CPN50) was prepared to study how salt concentration affects the structural, thermal, and electrochemical properties of the electrolyte system. Spectroscopic and structural analyses confirmed strong interactions between the polymer matrix and salt, accompanied by an increase in the amorphous character, which is favorable for ion transport. Thermal studies further indicated composition-dependent thermal behavior and suitable stability over the investigated temperature range. Among the investigated compositions, the electrolyte containing 30 wt% NaClO_4_ (CPN30) showed the best performance, exhibiting bulk conductivity of 259.55 ± 19.88 µS cm^−1^, with an electrochemical stability window of 2.6 V, and an ionic transference number (0.9988), indicating dominant ionic conduction. The CPN30 electrolyte was integrated into symmetric activated carbon (AC) electrodes and laser-induced graphene (LIG) electrodes. The AC-based device showed a specific capacitance of 19.65 F g^−1^ at 1 mV s^−1^ from cyclic voltammetry (CV) and 19.01 F g^−1^ at the current density of 0.09 A g^−1^ from galvanostatic charge–discharge (GCD) analysis. The flexible LIG-based device achieved an areal capacitance of 22.31 mF cm^−2^ at 1 mV s^−1^ from CV and 19.94 mF cm^−2^ at the current density of 0.0225 mA cm^−2^ from GCD. Following this, mechanical flexibility tests demonstrated that the LIG-based device retained approximately 95.00% of its capacitance from the bending angle 180° to 90° and 92.56% after 500 bending cycles. In addition, after 3000 cycles, the device retained 87.52% of its initial capacitance, with a coulombic efficiency of 91.42%. These results suggest that NaClO_4_-doped chitosan-PEG electrolytes are promising candidates for sustainable, flexible solid-state energy storage applications.

## Abstract 2

Laser-induced graphene (LIG) electrodes integrated with sustainable solid polymer electrolytes provide a flexible and eco-friendly platform for supercapacitor applications, offering improved charge storage performance and electrochemical stability.

## Graphical abstract

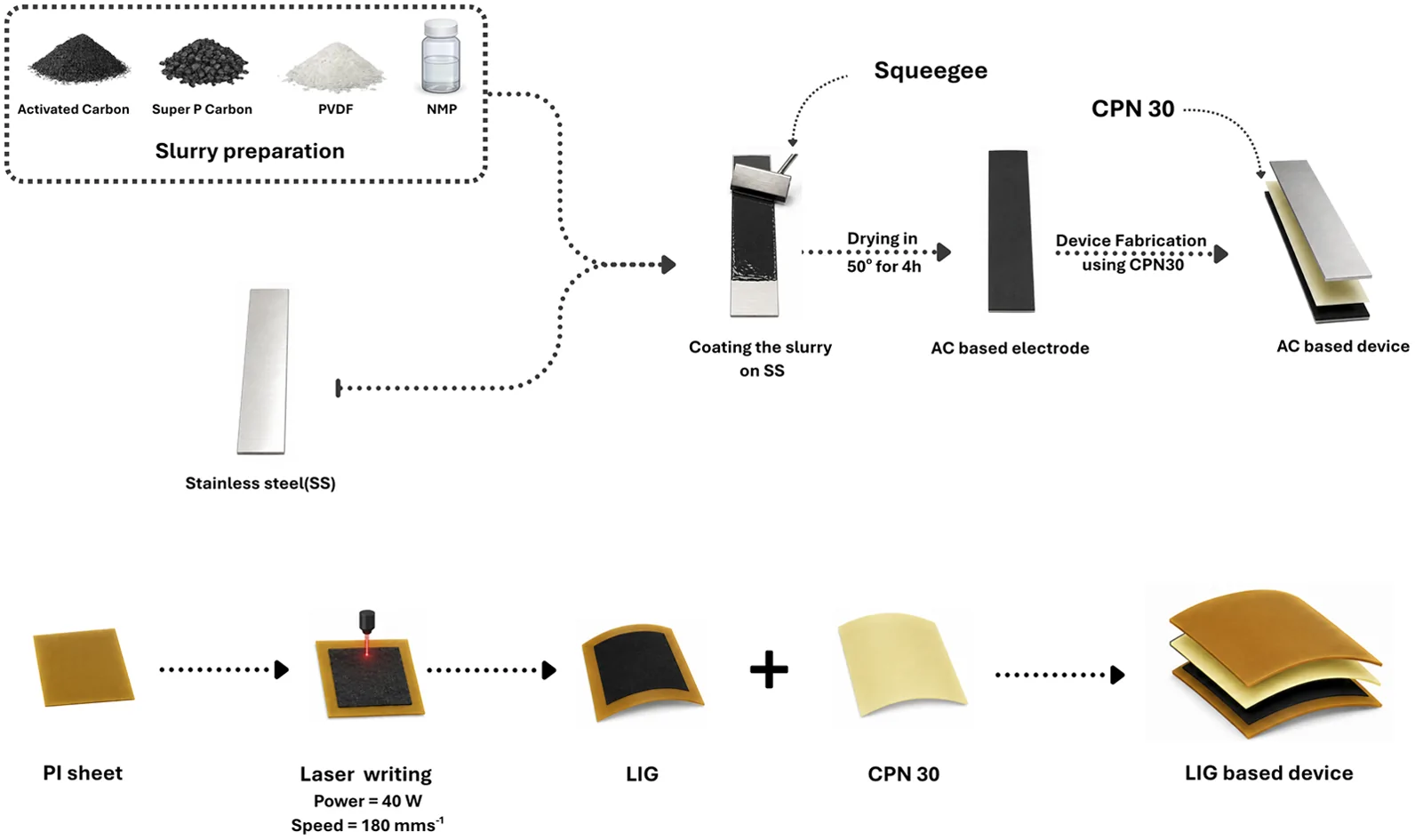

## 1. Introduction

The increasing demand for compact and flexible electronic gadgets has significantly influenced the evolution of energy storage technologies.^1^ This demand has driven advancements in various flexible energy storage devices, including batteries and supercapacitors, which are essential for wearable and portable electronics.^2,3^ Conventional supercapacitors based on liquid electrolytes, although electrochemically efficient, face practical challenges such as leakage, flammability, and integration difficulties with miniaturized and flexible systems.^4^ These limitations have driven considerable interest in all-solid-state supercapacitors, which offer improved safety, better mechanical stability, and easier integration into wearable and portable devices.^5^ The overall performance of an all-solid-state device is not determined by the electrode or the electrolyte alone, but rather by how effectively they interact within the system's confined architecture.^6^ In particular, the design of solid polymer electrolytes (SPEs) remains a critical factor, as they must simultaneously provide sufficient conductivity, mechanical flexibility, and long-term stability.^7^ At the same time, environmental sustainability has become an equally important consideration in material selection.

Biopolymer-based electrolytes are attractive alternatives to conventional petroleum-derived systems. Chitosan (CS) has attracted attention due to its biodegradability, natural abundance, and functional groups that can coordinate with ionic species.^8^ However, pristine CS typically exhibits low ionic conductivity (*σ*_ion_) and limited mechanical compliance, which restricts its direct use in high-performance solid-state devices.^9^ Therefore, structural modification strategies are essential.^10,11^

Blending CS with poly(ethylene glycol) (PEG) provides an effective route to enhance polymer chain mobility and reduce crystallinity, thereby facilitating ion transport within the matrix.^12,13^ Additionally, incorporating suitable sodium salts, such as NaClO_4_, introduces mobile charge carriers, further improving the electrochemical characteristics of the system.^14^ Therefore, careful control of the polymer composition and salt concentration is necessary to achieve a balance between conductivity and mechanical integrity.^15^ NaClO_4_ was selected as the ionic salt source due to its good dissociation characteristics and ability to provide mobile Na^+^ ions for efficient ion transport within the polymer matrix.^16^ Furthermore, sodium-based salts are inexpensive, abundant, and attractive for the development of sustainable solid-state energy storage systems.^17^

The electrode framework employed in the device is equally important. Carbon-based materials, including activated carbon (AC) and laser-induced graphene (LIG), are widely used because of their high surface area and good electrical conductivity.^18^ LIG is particularly attractive for flexible applications because of its porous morphology, scalable fabrication process, and direct patterning on polymer substrates.^19,20^ The interconnected structure of LIG facilitates intimate contact with the SPE, which is crucial for efficient charge storage in all-solid-state configurations.

In this study, NaClO_4_-doped CS:PEG SPE were developed and integrated into both AC-based and flexible LIG-based all-solid-state supercapacitors. By systematically analysing the relationship between electrolyte composition, structural characteristics, ion transport behaviour, and device-level electrochemical performance, this study aims to provide deeper insights into the rational design of sustainable solid-state energy storage systems suitable for next-generation flexible electronics.

In our previous work,^21^ the CS:PEG polymer host was investigated using LiClO_4_ as the conducting salt. The choice of NaClO_4_ in the present study is motivated by the abundance, ready availability, and cost-effectiveness of sodium compared with lithium, making Na^+^ an attractive alternative charge carrier for sustainable electrochemical energy-storage applications. Although Li^+^-based polymer electrolytes have been extensively investigated, the systematic replacement of Li^+^ with Na^+^ within the same CS polymer host remains comparatively less explored. Therefore, this work presents a novel approach to replacing Li^+^ with Na^+^ to investigate the influence of cation substitution on polymer-salt interactions, structural characteristics, ion-transport behavior, and electrochemical performance within the CS host matrix. The effect of NaClO_4_ concentration on the structural, thermal, and electrochemical properties of the electrolyte is systematically examined, followed by evaluation in all-solid-state supercapacitor devices.

A distinctive feature of this work is the implementation of the same optimized biopolymer electrolyte in two different electrode/current collector architectures: AC coated on stainless steel and LIG from a polyimide (PI) sheet. This enables evaluation of electrolyte performance across conventional and flexible solid-state device platforms while maintaining a common electrolyte system. Furthermore, the successful integration of the NaClO_4_-doped CS:PEG electrolyte with porous LIG electrodes demonstrates its suitability for flexible energy-storage applications. The findings establish the potential of the developed electrolyte as a sustainable solid-state electrolyte and provide insights into the relationship between electrolyte design, electrode architecture, and device performance.

## 2. Methodology

### 2.1. Chemicals used

Chitosan (derived from crab shells with a degree of deacetylation of 75%) was purchased from Loba Chemie Ltd. Poly(ethylene glycol) (PEG, M_W_ = 8000 g mol^−1^) was obtained from S.D. Fine Chemicals. Analytical-grade sodium perchlorate monohydrate (NaClO_4_^.^H_2_O, M_W_ = 140.46 g mol^−1^), used to dope the blend solution to enhance conductivity, was sourced from Tokyo Chemical Industry. Activated Carbon (AC, Kuraray, surface area = 1602 m^2^ g^−1^, purity = 99.99%) was used as the electrode material for supercapacitor fabrication, while polyvinylidene fluoride (PVdF), used as a binder, was procured from Sigma-Aldrich. *N*-Methyl-2-pyrrolidone (NMP), used as the solvent, and Super P carbon black (CSP, conductive additive) were purchased from Thermo Fisher Scientific. Stainless steel foil (thickness = 80 µm) used as the current collector was obtained from a local vendor. A polyimide (PI) sheet with a thickness of 70 µm, procured from Dongguan LKT Electronic Tech Co., Ltd, was used for the preparation of LIG. Conductive silver paste (Shilpent), and self-adhesive copper tape (Euro) was used for device assembly.

### 2.2. Preparation of blend solution

The preparation of the CS:PEG blend was optimized in our previous work.^21^ Briefly, 0.35 g of CS was added to 1% acetic acid and stirred for 12 h. Then, 0.15 g of PEG was added to the solution, and the mixture was stirred for an additional 12 h.

### 2.3. Preparation of NaClO_4_ based CS:PEG solid polymer electrolyte

NaClO_4_ was added to the optimized blend solution to enhance the *σ*. NaClO_4_ was added at different weight ratios (10–50 wt% with respect to total mass of the polymer blend). The weights of the individual polymer and salt are given in Table S1. Each solution was stirred for 12 hours. It was then allowed to rest for 4 h. The homogeneous mixture was subsequently cast onto clean petri dishes and dried in a hot air oven at 50 °C for 12 h. Once thoroughly dried, the free-standing films were carefully removed and stored for further characterization. The NaClO_4_ concentration was restricted to 50 wt% (CPN50), since excessive salt loading can induce ion aggregation and ion-pair formation, thereby decreasing the availability of mobile charge carriers and reducing *σ*. The workflow of the preparation, optimization, and characterization is shown in Fig. 1.

**Fig. 1:**
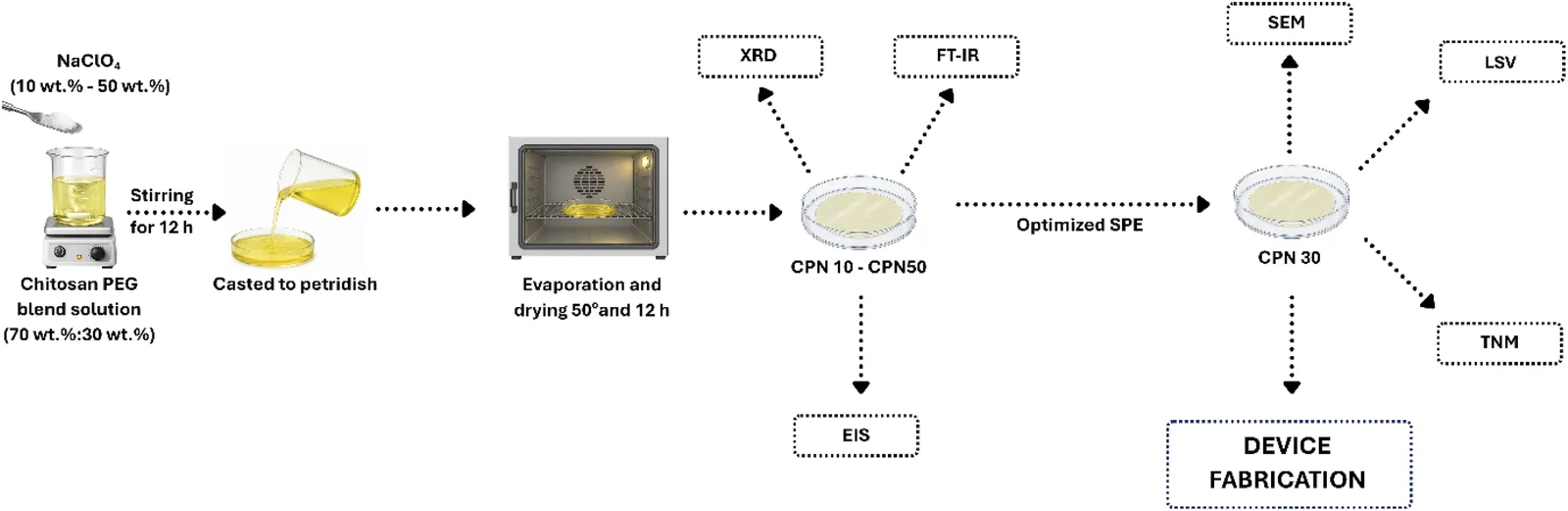
Preparation, optimization, characterization, and workflow of the CS:PEG:NaClO_4_ SPE.

### 2.4. Characterization of SPEs

The following sections outline the methods used to characterize the CS:PEG electrolytes modified with NaClO_4_. The characterization involved structural, thermal, surface morphology, and electrochemical analyses. The performance of the fabricated supercapacitor devices was evaluated to assess the practical applicability of the developed electrolytes.

#### 2.4.1. FT-IR

Fourier transform infrared (FT-IR) spectroscopy was used to investigate the structural characteristics and molecular interactions in the CS-PEG polymer matrix and its NaClO_4_-doped systems. The spectra were collected using a Shimadzu FT-IR Spirit QATR-S instrument in the Attenuated Total Reflectance (ATR) mode over the wavenumber range of 4000–400 cm^−1^, with a spectral resolution of 4 cm^−1^.

#### 2.4.2. XRD analysis

The crystalline and amorphous nature of the SPEs was examined using X-ray diffraction (XRD). Measurements were conducted on a Rigaku SmartLab high-resolution X-ray diffractometer employing Cu-Kα radiation (*λ* = 1.5406 Å). The diffraction data were collected over a 2*θ* range of 10°–80° at a scan rate of 4° min^−1^.

### 2.5. Thermal analysis

The thermal behavior and transition characteristics of the CS:PEG-based SPEs were examined using differential scanning calorimetry (DSC). The measurements were performed using a PerkinElmer DSC 6000 instrument operated using Pyris software. Samples weighing approximately 5 mg were heated from 30 °C to 400 °C at a heating rate of 10 °C min^−1^ under a continuous nitrogen atmosphere. Thermal stability and decomposition behavior were investigated using thermogravimetric analysis (TGA) and derivative thermogravimetry (DTG) on a PerkinElmer STA 6000 instrument controlled by Pyris software. The samples were heated from room temperature to 850 °C at 10 °C min^−1^ under a continuous nitrogen flow.

### 2.6. Morphological analysis

The surface and cross-sectional morphologies of the SPE films were examined using a Field Emission Scanning Electron Microscope (FE-SEM, Apreo 2 S, Thermo Fisher Scientific). The analyses were performed at room temperature to investigate the microstructural features and film morphology.

### 2.7. Conductivity measurement of SPEs

The thickness of the NaClO_4_-doped solid electrolyte films was determined using a micrometer screw gauge (Mitutoyo Micrometer, least count = 0.001 mm) to obtain accurate dimensional parameters for *σ* calculations. Electrochemical impedance spectroscopy (EIS) was used to evaluate the *σ* of the films using a Biologic SP-150 potentiostat. For the impedance measurements, the solid electrolyte film was sandwiched between two stainless-steel (SS) electrodes in a symmetric SS|SPE|SS configuration. The electrolyte film was sandwiched between SS electrodes with an effective electrode contact area of 1 cm × 1 cm. The measurements were carried out over a frequency range of 1 MHz to 100 mHz with an applied AC amplitude of 10 mV. The impedance data were represented as Nyquist plots (−Im(*Z*) *versus* Re(*Z*)) and fitted using an appropriate equivalent circuit model to determine the bulk resistance (*R*_b_), and was calculated using eqn (1):1
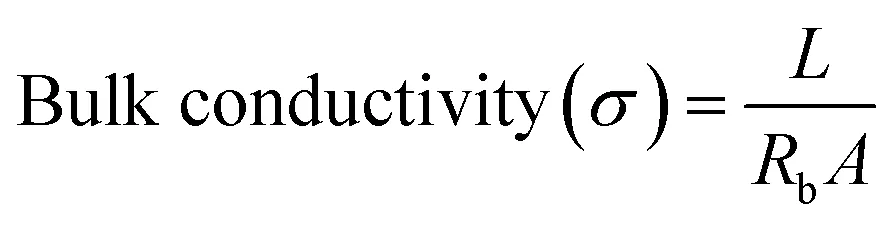
where *L* (cm) represents the thickness of the solid electrolyte film, *R*_b_ (Ω) is the bulk resistance obtained by fitting the Nyquist plot, and *A* (cm^2^) is the effective area of the electrolyte.

Each electrolyte composition was measured using ten independently prepared samples (*n* = 10). For each specimen, the corresponding film *L* and bulk resistance *R*_b_ were used to calculate the bulk conductivity (*σ*) individually according to eqn (1). The reported *R*_b_ and *σ* values are presented as mean ± standard deviation based on the ten independent measurements.

### 2.8. Electrochemical stability window analysis

Linear sweep voltammetry (LSV) was performed to determine the electrochemical stability window (ESW) using a symmetric cell configuration with the polymer electrolyte film sandwiched between two identical SS electrodes (SS|SPE|SS). The potential was swept linearly from 0 to 5 V at a scan rate of 10 mV s^−1^ using the same potentiostat.

### 2.9. Total ionic transference number study

Total transference number measurements (TNM) were performed at room temperature on the highest conducting SPE films using potentiostat. The films were assembled between two stainless steel electrodes in a symmetric SS|SPE|SS configuration. A constant DC voltage of 100 mV was applied for 1 h, and the resulting current was recorded over time to evaluate ion transport behavior and electrochemical characteristics of the SPEs. The ionic transference number *t*_ion_ was calculated using eqn (2):2
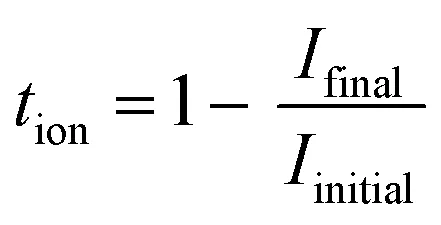
where, *I*_initial_, and *I*_final_ denote the initial and steady-state currents obtained from the DC polarization curve, respectively.

The ionic conductivity *σ*_ion_ was then calculated using eqn (3)

3 *σ*_ion_ = *σ*_bulk_ × *t*_ion_

where, *σ*_bulk_ represents the bulk conductivity obtained from EIS and *t*_ion_ represents the measured transference number.

### 2.10. Fabrication of the device and performance analysis

Supercapacitor devices were fabricated using AC-based electrodes. AC, CSP and PVDF binder were mixed in a weight ratio of 80 : 10 : 10. 150 µL of NMP was added to obtain a uniform slurry, which was then coated evenly onto a 1 cm × 1 cm SS substrate. The coated electrodes were dried overnight in an oven at 50 °C. Two identical electrodes were assembled in a symmetric configuration, with a CPN30 electrolyte film placed between them. A schematic diagram of the fabrication steps is shown in Fig. 2.

**Fig. 2:**
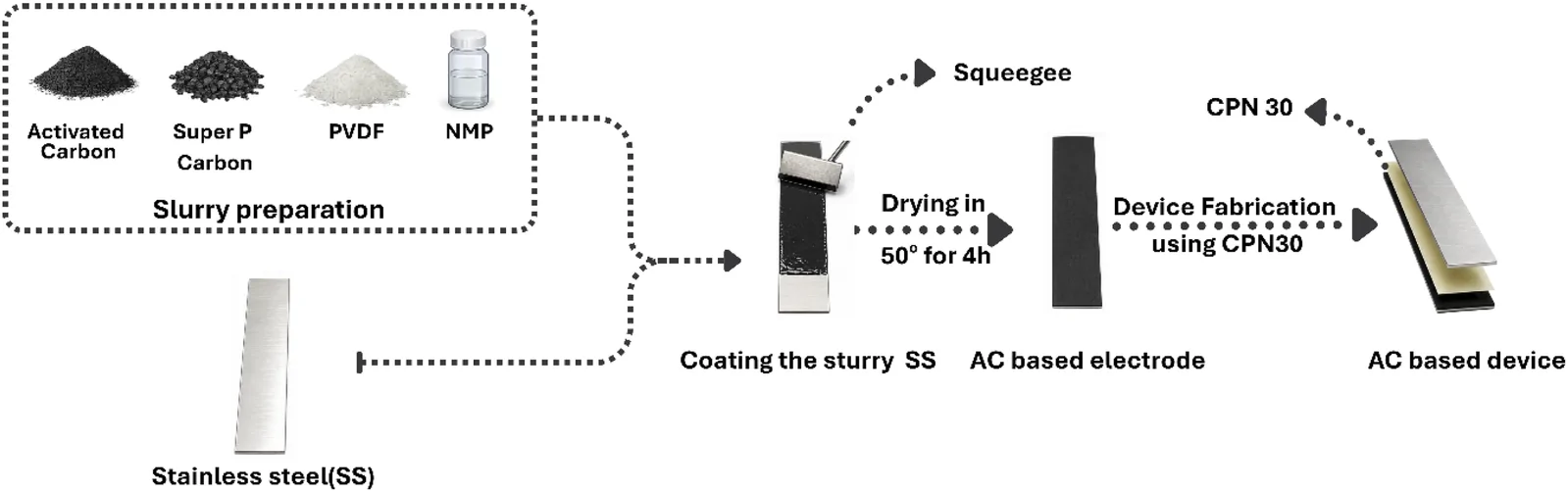
Schematic of the fabrication steps of the AC-based all-solid-state supercapacitor using the CPN30 SPE.

LIG was prepared using a laser power of 40 W and a scanning speed of 180 mm s^−1^ over a 2 cm × 2 cm area, following the previously reported fabrication method.^18^ The Raman characteristics of LIG obtained using this approach were also reported in the previously reported study. The characteristic D, G, and 2D bands were observed at approximately 1340, 1590, and 2700 cm^−1^, respectively, with *I*_D_/*I*_G_and *I*_2D_/*I*_G_ratios of 2.38 and 0.44, respectively. The electrical properties of the LIG used in the present study were evaluated at different positions across the electrode using the van der Pauw method with an Ossila four-point probe system. The measured sheet resistance, resistivity, and electrical conductivity were 15.14 ± 0.66 Ω sq^−1^, (1.060 ± 0.046) × 10^−3^ Ωm and 945.25 ± 40.31 S m^−1^ respectively. The LIG electrodes were assembled into a symmetric configuration with a CPN30 electrolyte between them. The schematic of the fabrication is shown in Fig. 3.

**Fig. 3:**
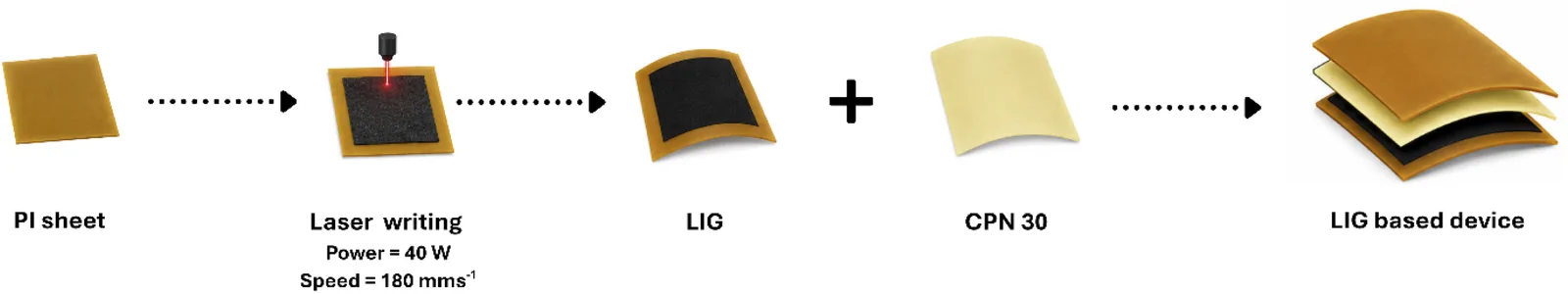
Schematic illustration of the fabrication process of a flexible LIG-based all-solid-state supercapacitor using CPN30 SPE.

The performance of the fabricated device was analyzed using CV, GCD, and EIS techniques. All electrochemical characterizations were performed using a Biologic SP-150 potentiostat. CV was performed at various scan rates in the potential window of 0 V to 1.3 V. For the AC-based device, the GCD characteristics were determined at current densities ranging from 0.09 to 1 A g^−1^. For the LIG-based device, GCD measurements were carried out at current densities ranging from 0.0225 to 0.25 mA cm^−2^. The EIS was performed from 1 MHz to 100 mHz using an AC voltage of 10 mV. Impedance fitting was performed using BioLogic EC-Lab software (BioLogic Science Instruments). The electrical equivalent circuits (EEC) used for the AC-based and LIG-based devices were selected according to the features observed in the corresponding Nyquist plots.

The data collected were used to calculate the electrochemical performance of a full symmetric two-electrode supercapacitor. The specific capacitance for AC based device was calculated using eqn (4) and (5), where the energy and power densities of the AC based device were calculated according to eqn (6) and (7), and the areal capacitance for the LIG based device was calculated using eqn (8) and (9), where the energy and power densities of the LIG based device were calculated according to eqn (10) and (11), respectively.^18,22^From CV,4
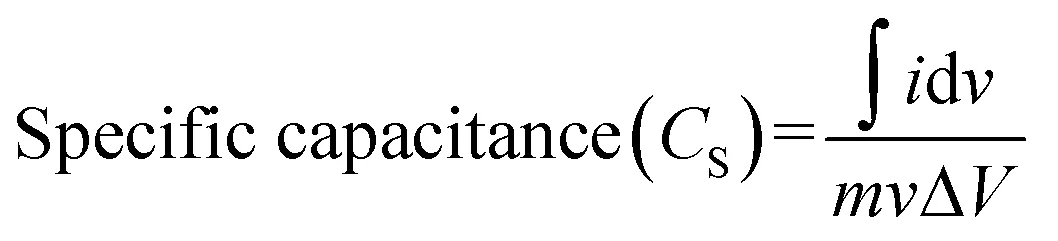
where ∫*i*d*v* is the area under the CV curve, *v* is the scan rate used in the study, *m* is the mass of the deposited electrode layer on one electrode (g), including AC, CSP, and PVDF (0.001 g), and Δ*V* is the potential window used.From GCD5
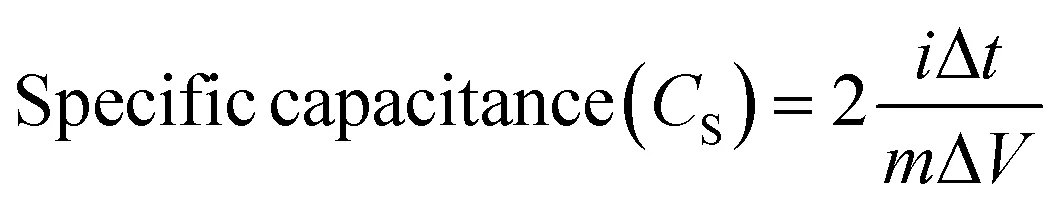
where *i* is the applied current (mA), Δ*t* is the discharge time excluding the IR drop,6
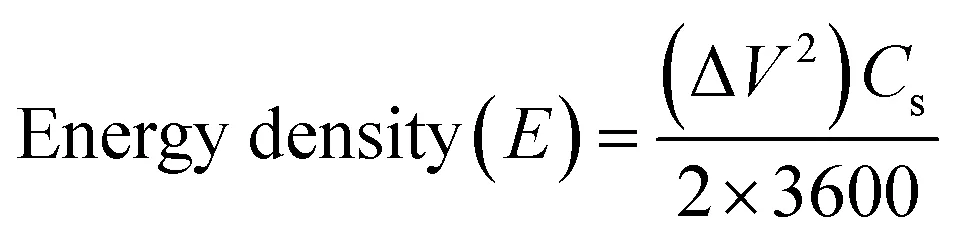
7
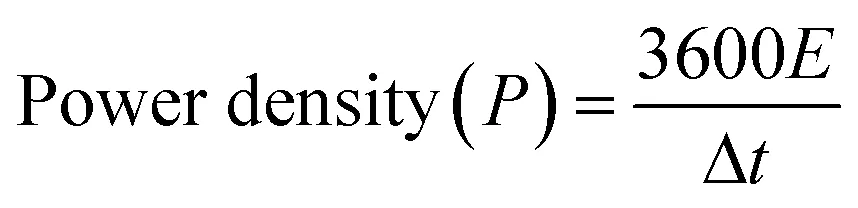
From CV8
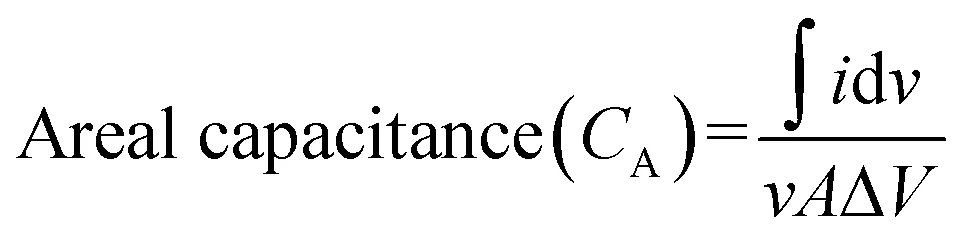
where ∫*i*d*v* is the area under the CV curve, *v* is the scan rate used in the study, *A* is the active area of the electrode and Δ*V* is the potential window used.From GCD 9
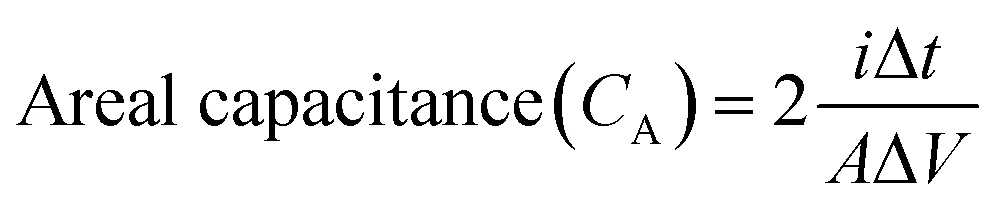
where *i* is the applied current (mA), Δ*t* is the discharge time excluding the IR drop,10
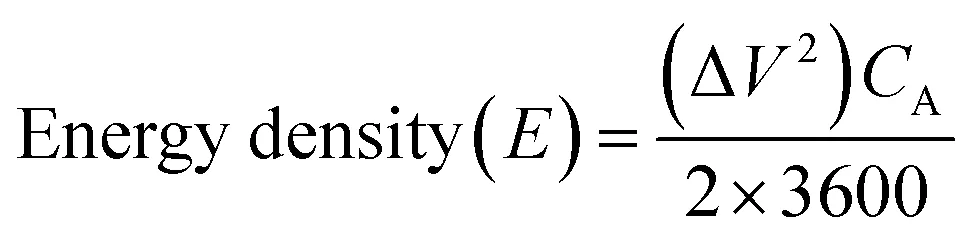
11
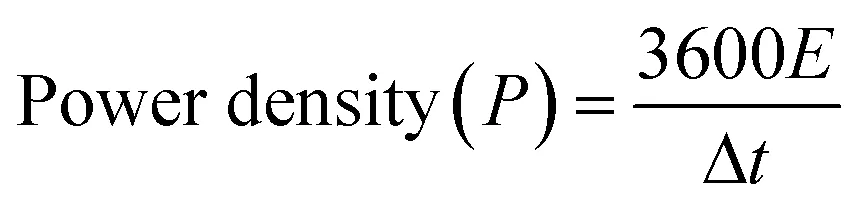


### 2.11. Mechanical and electrochemical stability test

The mechanical flexibility of the fabricated device was evaluated by subjecting it to repeated bending for up to 500 cycles. The electrochemical performance of the LIG-based device was evaluated at bending angles of 90° and 135° corresponding to bending radii of 0.69 cm and 2.55 cm respectively. For each configuration, the device was maintained at the respective bending angle during CV measurements to evaluate its electrochemical performance under mechanical deformation. The CV curves were recorded at a scan rate of 10 mV s^−1^. For the bending-cycle test, the device was repeatedly bent to approximately 90° and returned to its original unbent position at a rate of approximately 1.2 cycles per second (12 cycles in 10 s) for 500 cycles. GCD measurements were recorded before the bending test and after completion of the 500 bending cycles to evaluate the electrochemical stability of the device following repeated mechanical deformation.

The long-term cyclic stability of the AC-based device was evaluated by GCD cycling for 3000 cycles at a constant current density of 0.2 A g^−1^. The LIG-based device was subjected to 3000 GCD cycles at a constant current density of 0.125 mA cm^−2^. Capacitance retention and coulombic efficiency were evaluated throughout the cycling tests. Coulombic efficiency was obtained directly from the GCD cycling data using the EC-Lab software. The software calculates the coulombic efficiency for each cycle from the ratio of discharge time to charge time (equivalently, discharge capacity to charge capacity under constant-current conditions). The reported value corresponds to the coulombic efficiency at the 3000th charge discharge cycle. Capacitance retention was calculated from the GCD data, and the reported value corresponds to the capacitance retention at the 3000th cycle. These parameters were used to assess the durability and electrochemical stability of the AC- and LIG-based devices in combination with the CS:PEG:NaClO_4_ electrolyte.

## 3. Results and discussions

### 3.1. FT-IR

Structural modifications in the CS:PEG matrix upon the incorporation of NaClO_4_ were examined using FT-IR spectroscopy. The characteristic bands of CS, PEG, and salt were tracked to identify polymer-salt interactions and changes in the functional group environment. Fig. 4 shows the FT-IR spectra of the CPN series SPEs, and Table 1 lists the corresponding vibrational band assignments. Together, these illustrate the structural modifications in the polymer matrix upon salt incorporation.

**Fig. 4:**
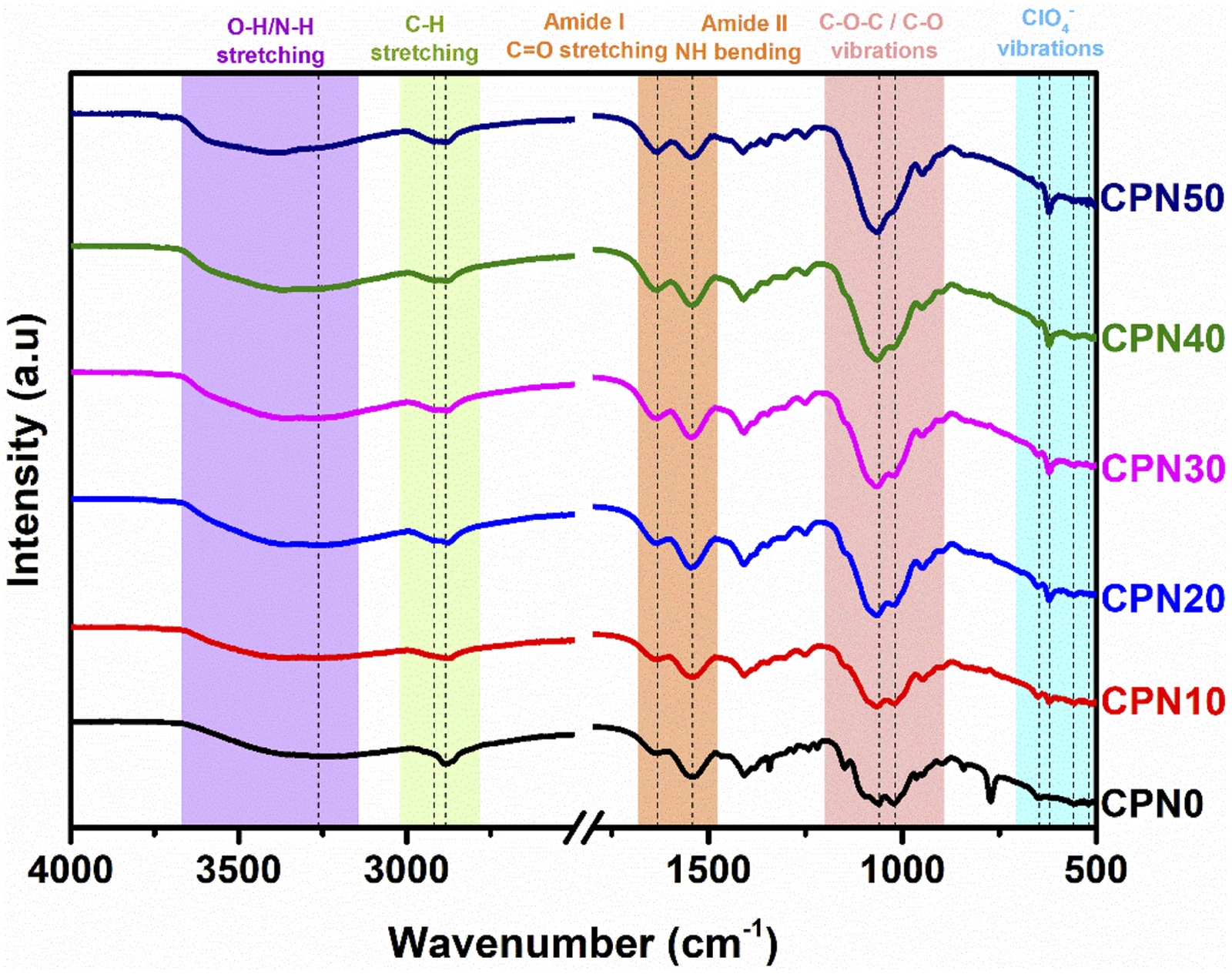
FT-IR spectra of CS:PEG:NaClO_4_ SPE (CPN0–CPN50) showing the effect of salt concentration on polymer-salt interactions and vibrational band variations.

**Table 1:** FT-IR band assignments of the CPN series with corresponding wavenumbers

Wavenumber (cm^−1^)	CPN0	CPN10	CPN20	CPN30	CPN40	CPN50	Inference
–OH/–NH stretch	3263	3262	3273	3273	3260	3278	Na^+^ disturbs H-bonding, consistent with salt–polymer interaction
C–H stretch	—	—	—	2917	2917	2917	Stable aliphatic backbone; weak effect at high salt
C–H stretch	2884	2882	2882	2882	2883	2883	Minor perturbations; reversible local chain vibrations
Amide I	1632	1632	1632	1632	1632	1631	Weak interaction of Na^+^ with amide C=O/N–H
Amide II	1542	1543	1546	1546	1546	1546	Backbone preserved; slight perturbation
CH_2_ bending	1468	—	—	—	—	—	Only visible in salt-free blend
CH deformation	1407	1409	1409	1409	1409	1412	Polymer chain rearrangement
CH/C–N bending	1343	1348	1348	1348	1349	1350	Na^+^ coordination with CS amine
C–N/OH mode	—	1304	1306	1306	1305	1304	Stable; minor backbone vibration
Amide III	1242	1251	1251	1251	—	1251	Confirms ionic interactions; suppressed at 40%
Backbone	1220	—	—	—	—	—	Present only in the salt-free blend
C–O–C	1149	—	—	—	—	—	Ether band of PEG; suppressed by salt
C–O–C stretch	1061	1066	1068	1068	1069	1067	PEG ether oxygens coordinate with Na^+^
C–O stretch	1022	1021	1022	1022	1022	—	Weak coordination with Na^+^
Skeletal	—	947	948	948	948	947	Stable structural vibration
β-linkage/ClO_4_^−^	899	—	—	—	831	832	Glycosidic bond suppressed; ClO_4_^−^ dominates at higher salt
ClO_4_^−^ deformation		647	650	649	649	—	Confirms salt incorporation
ClO_4_^−^ band	—	620	621	621	621	621	Diagnostic ClO_4_^−^-band; strong with salt
ClO_4_^−^ bending		559	556	556	556	556	Salt-anion interaction confirmed
ClO_4_^−^ vibration		520	513	514	514	512	Stable low-frequency ClO_4_^−^ vibration

The FT-IR spectra showed systematic changes in the characteristic absorption bands with increasing NaClO_4_ concentration, confirming strong interactions between the polymer matrix and the dissolved salt. The –OH/–NH stretching band shifted from 3263 cm^−1^ (CPN0) to 3278 cm^−1^ (CPN50), indicating that Na^+^ ions coordinate with the hydroxyl and amine groups of CS as well as the ether oxygen atoms of PEG. This coordination modifies the hydrogen-bonding network within the polymer matrix and alters the vibrational environment of these functional groups. The amide I and amide II bands exhibited only slight shifts, suggesting that the CS backbone remained largely intact while experiencing minor vibrations due to polymer–salt interactions. The gradual shift of the C–N and C–O–C stretching bands indicates coordination of Na^+^ ions with the amino groups of CS and the ether oxygen atoms (–O–) of PEG, providing additional evidence of polymer–salt complexation. The appearance of bands associated with ClO_4_^−^ ions in the lower wavenumber region further confirmed the successful incorporation of NaClO_4_ into the polymer matrix. The disappearance of some characteristic PEG and CS bands with increasing salt concentration was due to the overlap with perchlorate vibrations and changes in the local molecular environment caused by salt incorporation. Overall, these spectral changes demonstrate effective polymer–salt complexation, reduced intermolecular hydrogen bonding, enhanced amorphous character, and the formation of favorable pathways for Na^+^ ion transport.

FT-IR analysis confirmed that NaClO_4_ was successfully incorporated into the CS:PEG matrix. The Na^+^ ions interacted with the PEG oxygens and CS hydroxyl, amine, and amide groups, whereas ClO_4_^−^ ions contributed new vibrational features at lower wavenumbers. These findings established strong salt-polymer complexation, which is expected to play a crucial role in improving the *σ* of the prepared SPEs.^23–29^

### 3.2. XRD

The XRD patterns of the CS:PEG:based polymer electrolytes with different NaClO_4_ concentrations (CPN0–CPN50) are shown in Fig. 5. For the pristine blend (CPN0), a broad peak centered at 2*θ* ≈ 20°–23° is observed, which originates from the semicrystalline domains of PEG (typically reported at 19.2° and 23.3°) superimposed with the amorphous contribution of CS.^21,30^ This indicates that PEG retains partial ordering within the host matrix, while CS contributes to the halo-like background.

**Fig. 5:**
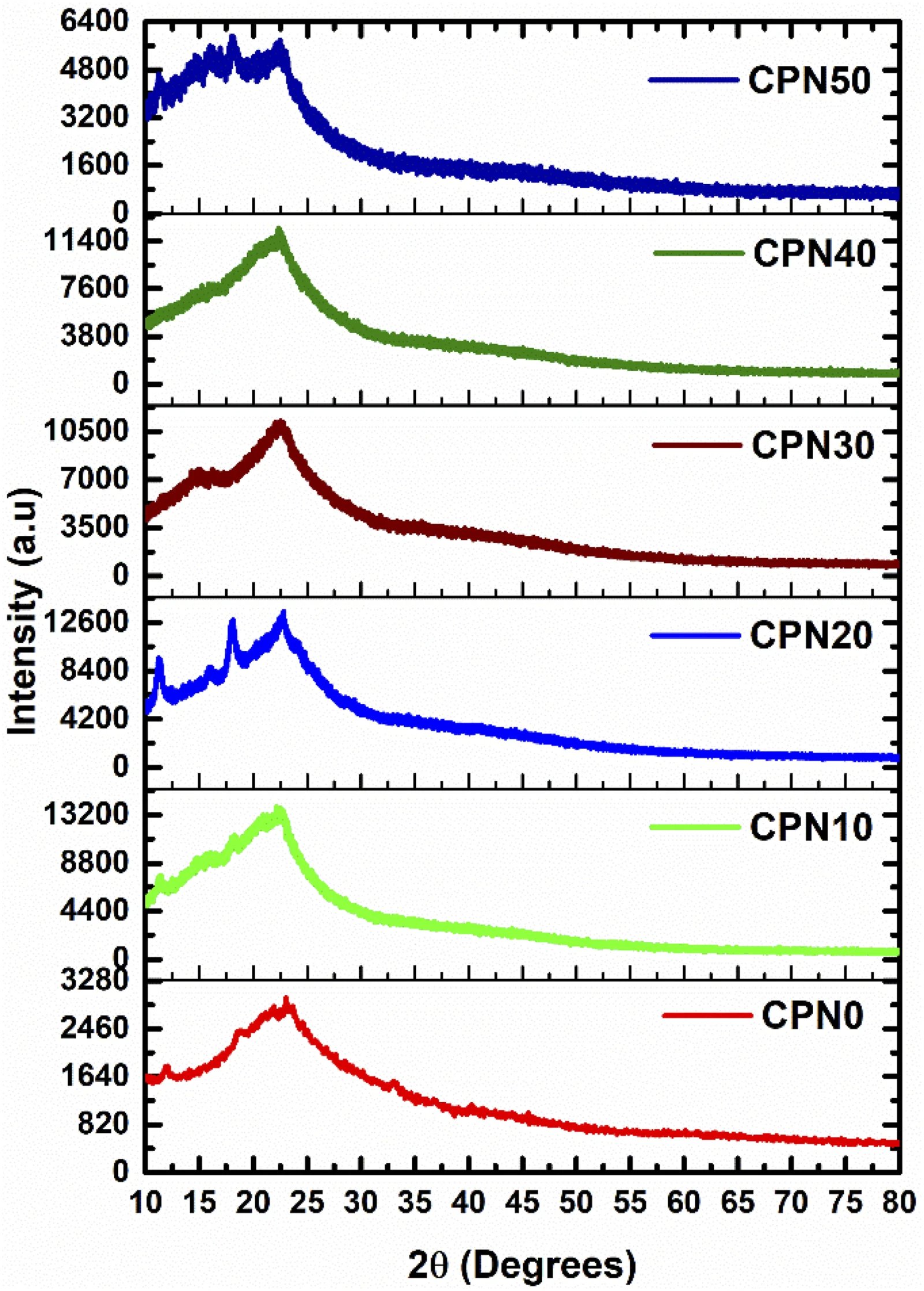
XRD patterns of CS:PEG:NaClO_4_ SPE (CPN0-CPN50) illustrating changes in crystallinity and amorphous nature with increasing NaClO_4_ concentration.

Upon the addition of NaClO_4_, the intensities of these PEG-associated peaks systematically decreased.^14^ In CPN10, the peak at 19°–20° becomes less intense and broadened, reflecting the initiation of polymer-salt complexation, in which Na^+^ ions coordinate with the ether oxygens of PEG and the amine/hydroxyl groups of CS. Simultaneously, the 23° reflection is suppressed, indicating disruption of the PEG chain packing. In CPN20, the halo around 20°–25° becomes even broader, and small shoulders emerge, which can be interpreted as a signature of microcrystalline salt-rich domains or local ordering induced by ionic aggregation.

This suggests that while the bulk polymer matrix becomes more amorphous, some regions accommodate the partially ordered complexes. For CPN30 and CPN40, the characteristic crystalline reflections of PEG almost disappeared, replaced by a dominant broad halo spanning 15°–30°, indicative of a predominantly amorphous structure. The absence of sharp Bragg peaks is consistent with suppressed long-range crystalline ordering, which may be associated with polymer–ion interactions. This structural transformation is advantageous for conductivity because the segmental mobility of the polymer backbone is enhanced in the amorphous state. Interestingly, in CPN50, weak and shallow peaks reappeared at approximately 20°–22°, which may reflect the emergence of salt-rich local ordering or partial recrystallization at high salt loading. These re-emerging crystalline features imply that high salt loading reduces the homogeneity of the electrolyte system, introducing ionic clusters that can hinder ion transport.

The systematic suppression of the PEG crystalline peaks at 19° and 23°, followed by the widening of the amorphous halo between 15° and 30°, is consistent with salt–polymer interactions and the associated reduction in crystalline ordering. The reappearance of shallow features at high loading highlights the delicate balance between salt concentration and amorphous phase stability in determining the microstructural and electrochemical properties of CS:PEG:NaClO_4_ polymer electrolytes.

### 3.3. Thermal analysis

The DSC thermograms of the CPN series (Fig. S1) provide information on the thermal response of the polymer electrolyte system. The glass-transition temperatures were determined from the corresponding enlarged transition regions using the baseline-intersection method, as shown in Fig. S1(a–f). The pristine polymer (CPN0) exhibited a *T*_g_ of 50.5 °C, representing the inherent chain mobility and baseline flexibility of the host matrix. With the incorporation of 10 wt% additive, the *T*_g_ shifted upward to 52.3 °C, reflecting restricted polymer segmental motion due to enhanced intermolecular interactions. A similar effect was observed in CPN20, where the *T*_g_ remained high at 52.0 °C, further suggesting that moderate additive levels reduce the free volume and stabilize the amorphous phase. Increasing the additive loading to 30 and 40 wt% resulted in a gradual decrease in *T*_g_ to 49.0 and 48.5 °C, respectively. This decrease suggests that, at these intermediate concentrations, the additive disrupts the polymer-chain packing, leading to increased local free volume and greater segmental mobility within the polymer matrix. However, when the content was increased to 50 wt%, *T*_g_ increased sharply to 56.8 °C, the highest value across the series, highlighting that stronger interfacial interactions dominate at higher concentrations and significantly stiffen the polymer backbone. The *T*_g_ values determined are tabulated in Table S2.

No distinct melting endothermic peak (*T*_m_) was observed in any of the CPN compositions before the onset of thermal decomposition. This behavior may be associated with the predominantly amorphous nature of the polymer electrolyte system and the suppression of crystalline ordering due to polymer–salt interactions. The absence of a well-defined melting endotherm therefore prevented the reliable determination of *T*_m_ for the investigated compositions.^31–34^

Importantly, all samples in the CPN series exhibited a single *T*_g_, with no evidence of multiple transitions. This observation is consistent with reasonable compatibility between the additive and host polymer components across the investigated compositions, with no apparent evidence of macroscopic phase separation. The observation of a single resolved *T*_g_ is consistent with apparent component compatibility on the DSC measurement scale, although it does not exclude finer-scale compositional heterogeneity. The non-linear trend of *T*_g_ therefore captures the delicate balance between two competing effects: chain restriction due to additive–polymer interactions and plasticization at intermediate concentrations. From a practical standpoint, this ensures that the CPN series retains amorphous uniformity while offering tunable flexibility and stability. Such a balance is crucial for SPE, where maintaining a homogeneous single-phase matrix with a tailored *T*_g_ is directly linked to ionic transport, electrochemical stability, and the mechanical robustness of energy storage devices.

The thermal stability of the CPN series was examined through TGA and DTG curves (Fig. 6a–f), which show the thermal response of the materials at increasing temperatures. In all samples, the black curves represent the gradual weight loss, while the red curves represent the rate of weight loss.

**Fig. 6:**
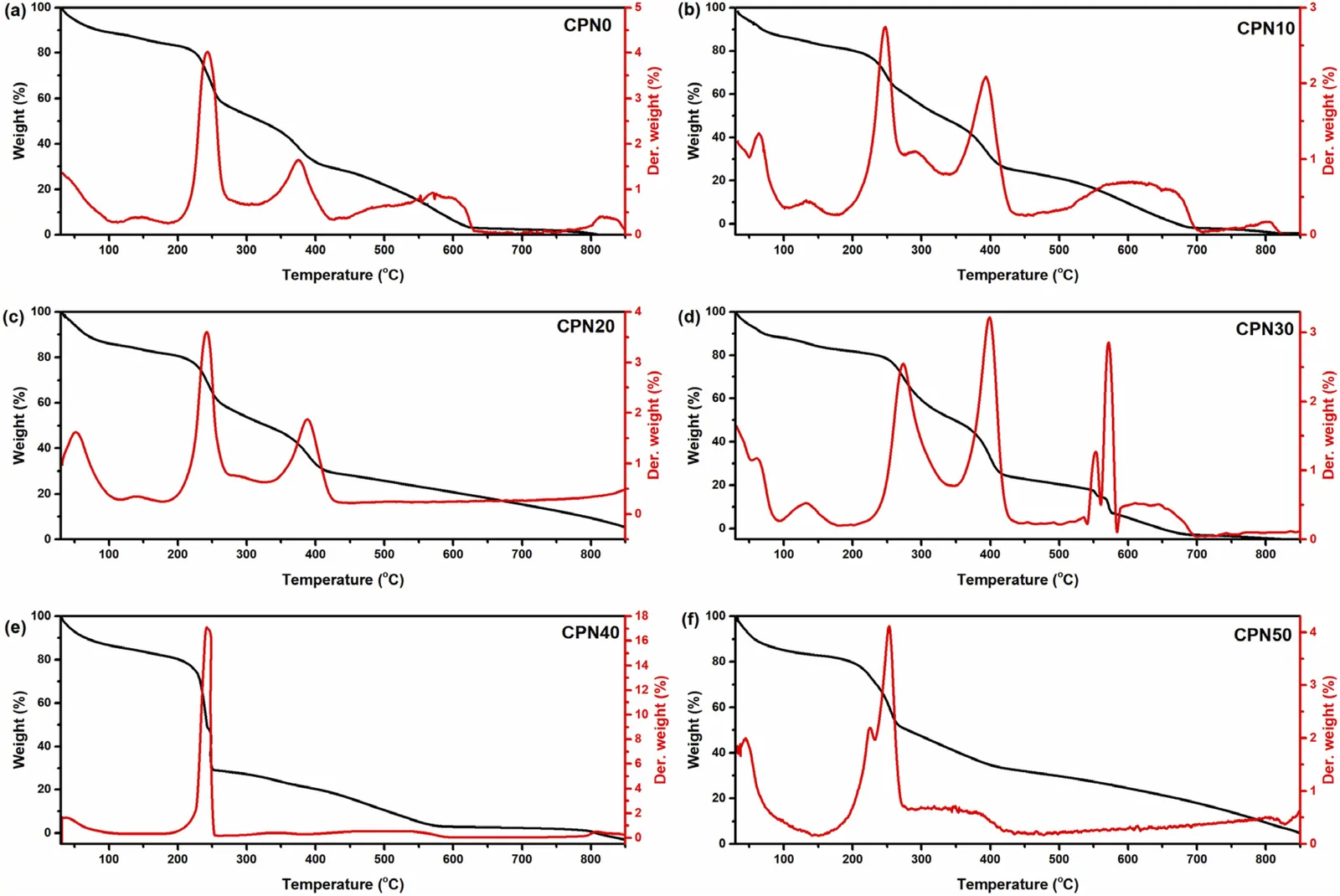
TGA (black) and DTG (red) curves of CS:PEG:NaClO_4_ SPE: (a) CPN0 (b) CPN10 (c) CPN20 (d) CPN30 (e) CPN40 and (f) CPN50.

For the pure polymer, CPN0 (Fig. 6(a)), three distinct stages of degradation were observed. The initial slight weight loss below 120 °C was due to evaporation of residual moisture or trapped solvents. The significant weight loss occurred between 200 °C and 400 °C, where the polymer backbone began to break down. Beyond 500 °C, further decomposition was associated with more stable fragments and finally, the breakdown of carbonaceous residue. In the doped samples CPN10 (Fig. 6(b)) and CPN20 (Fig. 6(c)), the degradation pattern remained multi-step, but the start of decomposition shifted slightly. The DTG peaks became sharper, indicating stronger interactions between the additive and the polymer, temporarily improving thermal resistance. This suggests that small amounts of additives reinforce the material without disturbing its overall homogeneity. In CPN30 (Fig. 6(d)), the degradation became more complex, with multiple distinct DTG peaks appearing between 250 °C and 600 °C. These features indicate multiple overlapping decomposition processes associated with the different components of the polymer electrolyte system. The increased complexity of the DTG profile reflects composition-dependent thermal behavior at this salt concentration.

In the case of CPN40 (Fig. 6(e)), the thermogram shows a distinct weight-loss step occurring around 250°–300 °C, followed by degradation at higher temperatures. The DTG peak is sharper than that of the other compositions, suggesting that at this salt concentration, the polymer–salt interaction shifts the stability window. For CPN50 (Fig. 6(f)), the thermal profile indicates multiple degradation stages extending to approximately 700 °C, with broad DTG features suggesting overlapping decomposition processes. The TGA measurement continued up to 850 °C, where residual mass remained, indicating incomplete volatilization and the contribution of inorganic salt residues. The broadened peaks and altered weight-loss behavior may be associated with changes in salt distribution and overlapping decomposition processes at higher salt loading. The persistence of residual weight at higher temperatures further supports the presence of thermally stable inorganic residues.

In summary, the TGA/DTG study showed a delicate balance: at low to moderate additive contents, the material gained some stability; at intermediate levels (30–40 wt%), it becomes less stable due to disrupted packing; at higher loading (50 wt%), the broader degradation profile and residual mass may be associated with localized salt-rich environments and overlapping decomposition processes and overlapping decomposition processes within the polymer matrix. Taking together with the DSC results, these findings suggest that the thermal behavior of the CPN series is highly tunable, and that an optimal composition can be selected to balance flexibility with the thermal endurance required for solid-polymer electrolyte applications.

### 3.4. Conductivity measurements

The Nyquist plots of the CPN series electrolytes are shown in Fig. 7(a)–(e). All compositions exhibited predominantly inclined responses without a well-defined high-frequency semicircular region.^35^ Therefore, the bulk resistance (*R*_b_) was not estimated from the diameter of a semicircle; instead, it was obtained from equivalent-circuit fitting using the *R*_b_ + *Q*1 model. In this model, *R*_b_ corresponds to the bulk/ohmic resistance of the SPEs, while *Q*1 represents the non-ideal capacitive response. The equivalent circuit model is shown in Fig. 7(f).

**Fig. 7:**
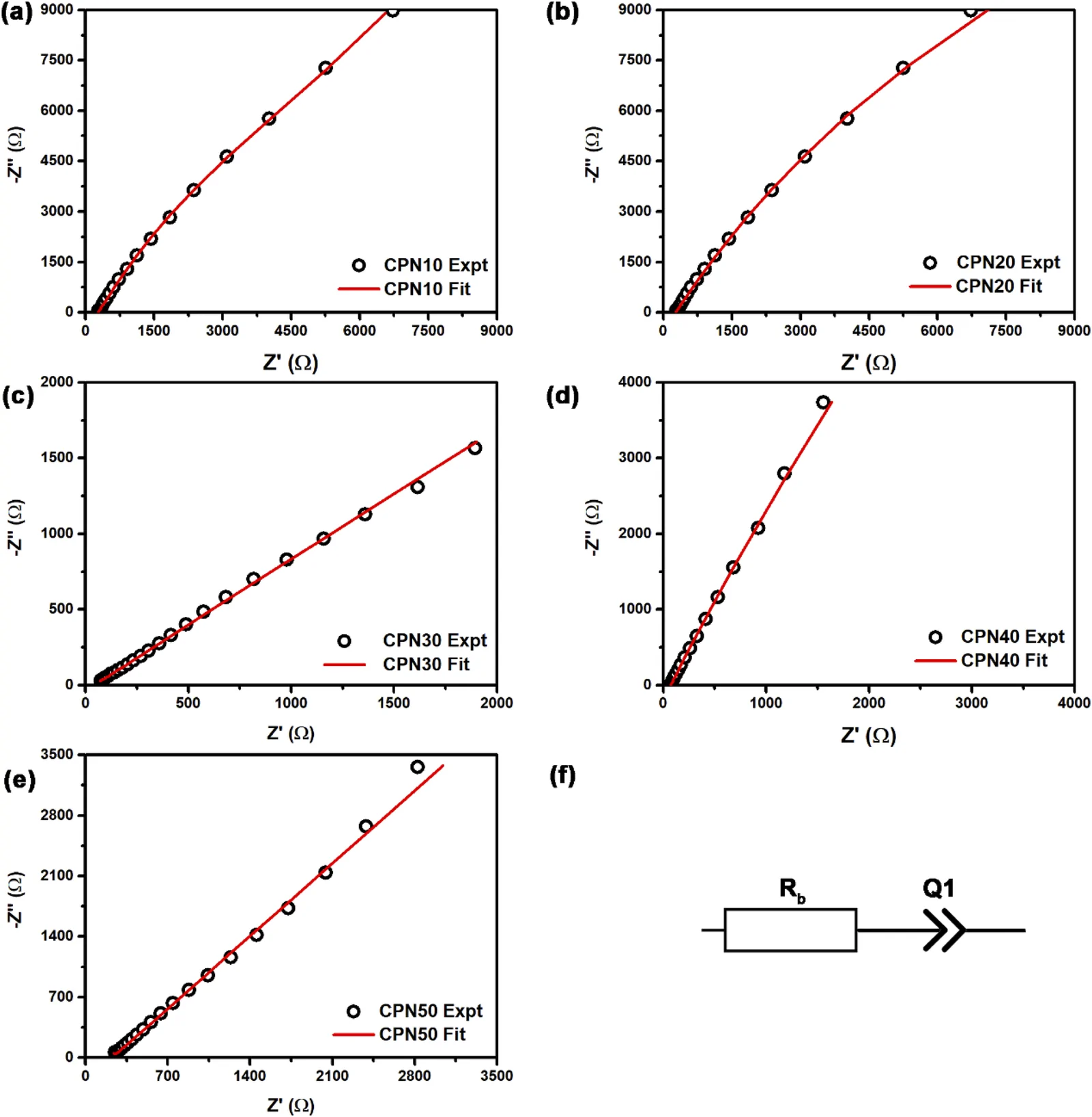
Nyquist plots of CS:PEG:NaClO_4_ SPEs with experimental data (symbols) and equivalent circuit fitting (solid lines): (a) CPN10, (b) CPN20, (c) CPN30, (d) CPN40, (e) CPN50, and (f) equivalent circuit model used for fitting the impedance data.

The *R*_b_ values extracted from the fits revealed a distinct composition-dependent behavior. The reported *R*_b_ and *σ* values correspond to the average of ten independent measurements (*n* = 10), and the error values represent the standard deviations.

The goodness-of-fit parameters obtained from the equivalent-circuit fitting are summarized in Table S3. The normalized *χ*^2^/∣*Z*∣ values provide a quantitative measure of the agreement between experimental impedance data and the fitted *R*_b_ + Q1 model. The relatively low normalized *χ*^2^/∣*Z*∣ values indicate good agreement between the experimental and fitted impedance responses. The fitted *R*_b_ values were subsequently used for the calculation of bulk conductivity. CPN10 showed the highest *R*_b_ (888 ± 25 Ω) and, correspondingly, the lowest *σ* (9.21 ± 0.26 µS cm^−1^). As the composition evolved to CPN20, the resistance dropped sharply to 267 ± 2.3 Ω, with an *σ* of 41.25 ± 0.36 µS cm^−1^. The best performance was observed for CPN30, where *R*_b_ reached a minimum of 41 ± 3.2 Ω and *σ* peaked at 259.55 ± 19.88 µS cm^−1^. Beyond this point, however, the *σ* decreases again: CPN40 and CPN50 exhibit *R*_b_ values of 69 ± 2.9 Ω and 228 ± 2.5 Ω, giving conductivities of 143.68 ± 6.12 µS cm^−1^ and 46.61 ± 0.51 µS cm^−1^, respectively. Thus, CPN30 exhibited the optimal formulation in this series, achieving the lowest resistance and highest *σ*.

The increase in *σ* from CPN10 to CPN30 is attributed to enhanced NaClO_4_ dissociation and increased amorphous character of the polymer matrix, which facilitates Na^+^ ion transport. The maximum conductivity observed for CPN30 reflects an optimum balance between charge-carrier concentration and ion mobility. Beyond CPN30, the *R*_b_ increases, and the *σ* decreases (Table 2).

**Table 2:** *R*_b_ and *σ* of CPN series SPEs (mean ± standard deviation, *n* = 10)

Name	Thickness (µm)	*R*_b_ (Ω)	*σ* (µS cm^−1^)
CPN10	82 ± 4	888 ± 25	9.21 ± 0.26
CPN20	110 ± 2	267 ± 2.3	41.25 ± 0.36
CPN30	109 ± 5	41 ± 3.2	259.55 ± 19.88
CPN40	100 ± 2	69 ± 2.9	143.68 ± 6.12
CPN50	106 ± 3	228 ± 2.5	46.61 ± 0.51

At higher NaClO_4_ concentrations (CPN50), the number of salt ions exceeds the coordination capability of the polymer matrix. This may lead to ion aggregation and the formation of ion pairs or ionic clusters. These neutral or weakly mobile species reduce the concentration of free Na^+^ ions available for conduction. In addition, the higher salt content restricts polymer segmental motion by reducing chain flexibility, thereby decreasing ion mobility through the polymer network. The combined effects of reduced charge-carrier mobility and increased ionic aggregation result in the higher *R*_b_ observed for CPN50.^36,37^

Because CPN30 exhibited the highest *σ* and enhanced amorphous nature among the investigated compositions, it was selected for further characterization and device fabrication studies.

### 3.5. Morphological analysis

The surface morphology of the optimized CPN30 SPE film was examined using FE-SEM, and the corresponding surface and cross-sectional micrographs are shown in Fig. 8. As shown in Fig. 8(a), the electrolyte film exhibits a relatively smooth and continuous surface with uniformly distributed micropores throughout the polymer matrix. The observed porous morphology may increase in an ion-accessible interfacial area and facilitate electrolyte–electrode contact; direct ion-transport pathways cannot be established from SEM alone. No obvious salt agglomeration or phase separation was observed, indicating effective polymer–salt complexation and homogeneous dispersion of NaClO_4_ within the CS/PEG matrix. The cross-sectional FE-SEM image (Fig. 8(b)) confirms the formation of a continuous and compact electrolyte film with a porous internal structure, uniform thickness, and no visible cracks or delamination. These observations demonstrate the good film-forming ability and mechanical integrity of the CPN30 electrolyte film. Such a homogeneous morphology is expected to facilitate efficient Na^+^ ion transport while maintaining structural stability during electrochemical operation, thereby demonstrating the suitability of the CPN30 SPE for all-solid-state supercapacitor applications.^38–40^

**Fig. 8:**
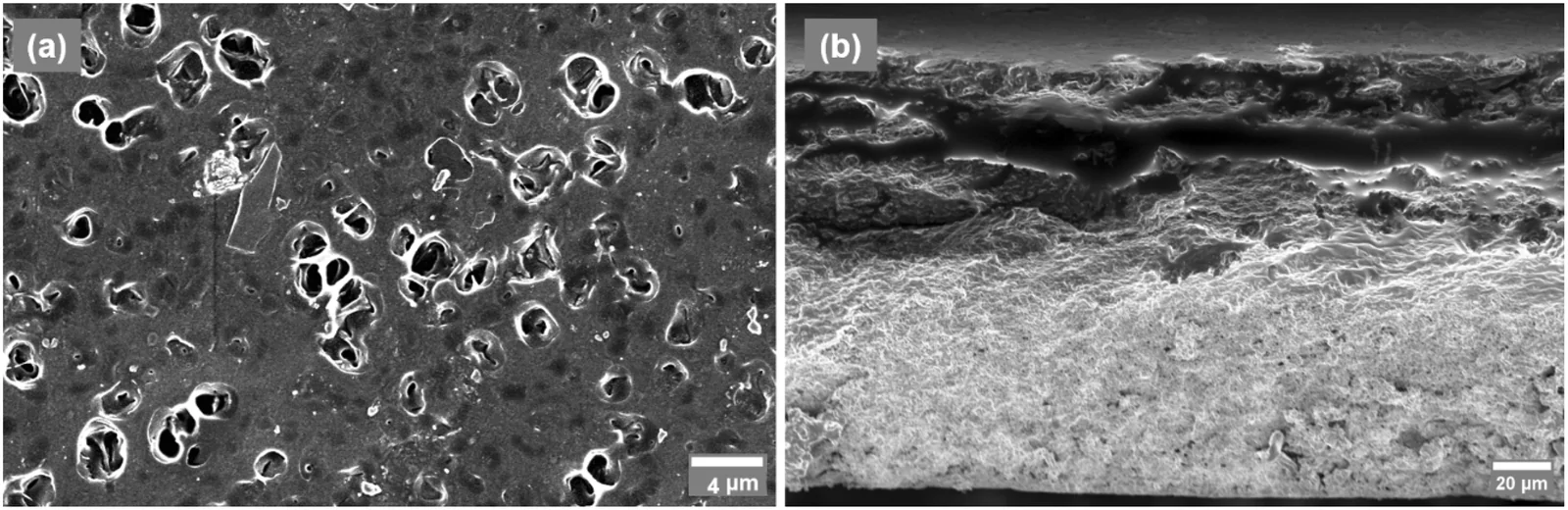
FESEM images of the CPN30 thin film showing (a) the surface morphology (top view) and (b) cross-sectional structure.

### 3.6. LSV

The ESW is a critical parameter that determines the applicability of polymer electrolytes in energy-storage devices. Fig. 9(a) shows the LSV curve of the CPN30 electrolyte system recorded at a scan rate of 10 mV s^−1^. The current remains negligible at lower potentials, indicating good electrochemical stability and minimal parasitic reactions.^41^ A sharp rise in current is observed above 2.6 V, attributed to the onset of electrolyte decomposition. Based on the intersection of the tangent lines drawn along the baseline and the rapidly increasing current region, the ESW of the electrolyte was estimated to be approximately 2.6 V. The LSV measurement performed using the SS|CPN30|SS configuration showed an electrolyte decomposition onset at approximately 2.6 V. This value represents the electrochemical stability of the electrolyte under the LSV conditions and should not be considered the practical operating voltage of the complete supercapacitor device.

**Fig. 9:**
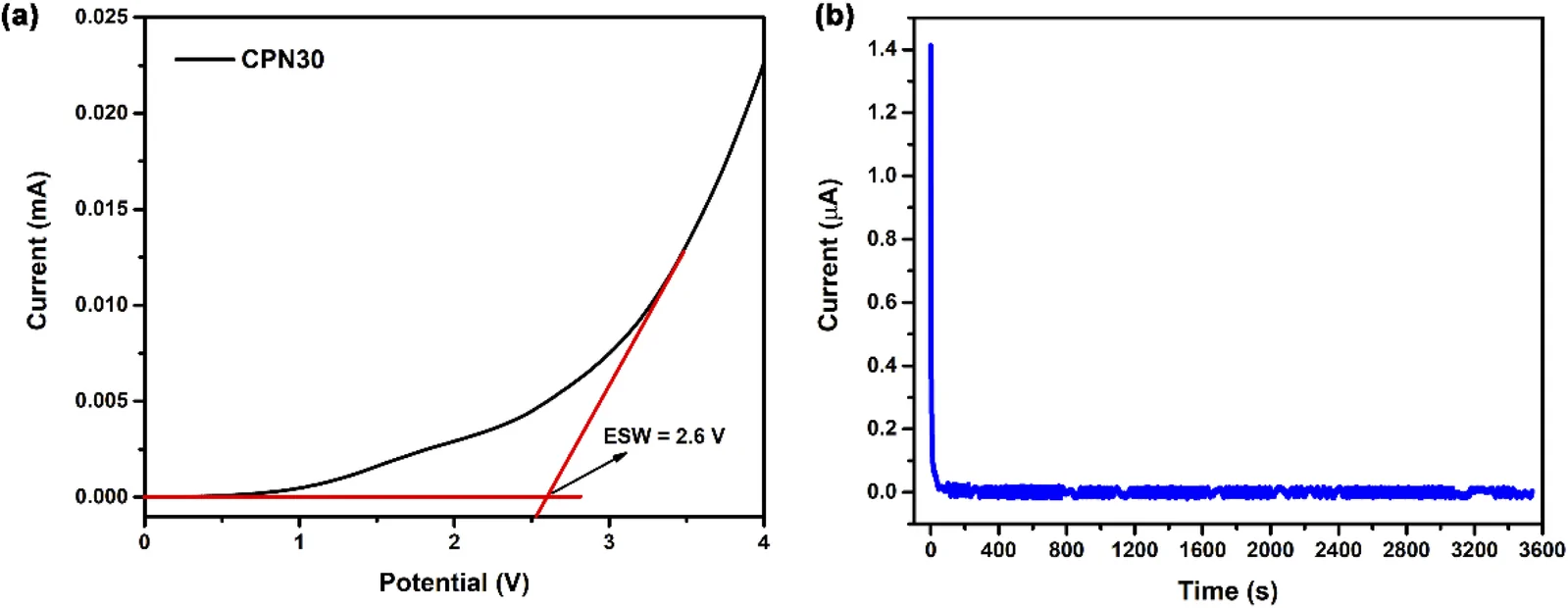
(a) LSV curve of the CPN30 electrolyte showing the ESW and (b) DC polarization curve of CPN30 recorded at a potential of 100 mV.

### 3.7. Total ionic transference number measurement

Fig. 9(b) shows the TNM graph. The measurement was performed by applying a constant DC polarization voltage of 100 mV across the SS|CPN30|SS cell for 3600 s. The current initially exhibited a value of 1.413 µA, which rapidly decreased with time and finally stabilized at a steady-state value of 0.00169 µA. TNM was calculated and it is found as 0.9988. The initial current is attributed to ions (cation and anion combined) and electrons, whereas the final current is only due to electrons. The rapid decay in current is attributed to electrode polarization and suppression of electronic conduction at the electrode–electrolyte interface. The extremely low steady-state current confirms that charge transport in the electrolyte is predominantly ionic, with negligible electronic contribution.

Based on the obtained TNM (0.9988), the ionic and electronic transference numbers correspond to 0.9988 and 0.0012, respectively. Considering the total conductivity measured, the ionic conductivity was determined to be 259.24 ± 19.86 µS cm^−1^, while the corresponding electronic conductivity was estimated to be 0.311 ± 0.024 µS cm^−1^. The substantially higher ionic conductivity compared with the electronic conductivity further confirms the predominantly ionic nature of charge transport in the SPE.

### 3.8. Electrochemical activity studies of AC-based device

The electrochemical performance of the AC-based device with CPN30 electrolyte (AC|CPN30|AC) was evaluated using CV, GCD, and cycling stability studies, as discussed in the following sections.

#### 3.8.1. CV analysis

Fig. S2 shows the CV curves obtained at different voltage windows ranging from 0–1.0 V to 0–1.5 V. In the 0–1.0 V range, the CV curves maintained a nearly symmetric and rectangular shape, indicating ideal electrostatic double-layer capacitive behaviour with a minimal faradaic contribution.^42^

As the potential window was expanded beyond 1.2 V, the current response increased significantly owing to the larger energy window for charge storage. However, a slight deviation from ideal rectangularity was observed at the widest range (0 to 1.5 V). This deviation is attributed to polarization effects and the onset of minor side reactions. However, AC-based device exhibits good electrochemical stability and reversibility up to approximately 1.3 V, which can be considered as its optimal operating window for stable energy storage. The 1.3 V operating window was selected based on the CV response of the AC-based device, which showed a stable current response without noticeable parasitic reactions and was therefore considered more suitable for prolonged device operation.

The effect of scan rate on the capacitive behavior was further explored in the range of 1 mV s^−1^ to 100 mV s^−1^, as shown in Fig. 10(a). At lower scan rates (1 mV s^−1^ to 10 mV s^−1^), the voltammograms retain their quasi-rectangular shape, reflecting efficient diffusion and full utilization of available active sites. At higher scan rates, the CV curves become progressively distorted and elongated, a phenomenon commonly attributed to limited ion mobility and increased polarization resistance during fast potential sweeps. Despite this, the symmetry of the curves suggests that AC-based device predominantly operates through non-faradaic charge storage and maintains good reversibility even under kinetic limitations.^43^

**Fig. 10:**
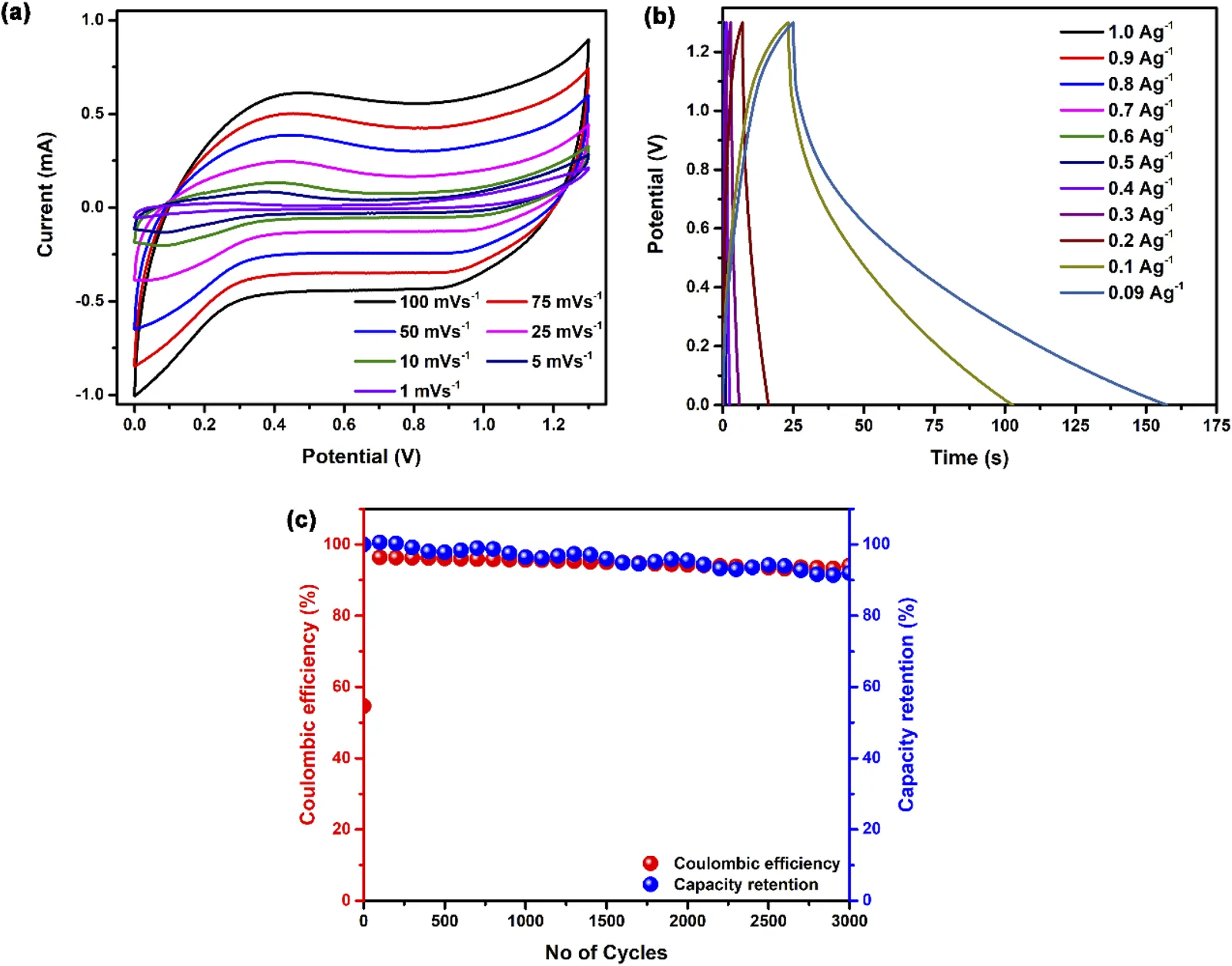
Electrochemical performance of the AC-based supercapacitor with CPN30 electrolyte: (a) CV curves recorded at different scan rates, (b) GCD profiles recorded at different current densities, and (c) cycling stability showing capacitance retention and coulombic efficiency over 3000 cycles.

The corresponding specific capacitance values calculated from the CV curves are summarized in Table 3. At the slowest scan rate of 1 mV s^−1^, AC-based device exhibited a maximum capacitance of 19.65 F g^−1^, reflecting the ability of ions to access the electrolyte–electrode interface fully. With increasing scan rate, the capacitance gradually decreased, reaching 10.17 F g^−1^ at 5 mV s^−1^, 8.21 F g^−1^ at 10 mV s^−1^, and finally dropping to 4.56 F g^−1^ at 100 mV s^−1^. This reduction is consistent with diffusion-controlled transport limitations at faster sweep rates, in which only surface-accessible sites contribute to charge storage.

**Table 3:** Specific capacitance of the device with AC based CPN30 at different scan rates calculated from CV curves

Scan rate (mV s^−1^)	Specific capacitance (F g^−1^)
100	4.56
75	4.92
50	5.49
25	6.55
10	8.21
5	10.17
1	19.65

Taken together, these observations highlight that AC-based device offers a favorable balance of electrochemical stability, capacitive reversibility, and ionic transport properties. The wide stability up to 1.3 V, combined with its high capacitance at low scan rates, underscores the potential of AC-based device as a promising SPE for energy storage applications, such as supercapacitors and solid-state batteries.

#### 3.8.2. GCD analysis

GCD measurements were performed at different current densities ranging from 0.09 to 1 A g^−1^ (Fig. 10(b)). The curves show the typical near-triangular shape expected for capacitive systems, confirming that charge storage is primarily electrostatic rather than redox.^44^ At lower applied current densities, such as 0.09 A g^−1^ to 0.2 A g^−1^, the discharge profiles are extended over longer timescales. This directly reflects a higher capacity for charge storage because ions have sufficient time to migrate, align, and occupy the available sites within the polymer matrix and at the electrode–electrolyte interface. The smooth, nearly linear voltage-time responses in this region highlight the uniform ionic motion and the stability of the electrolyte.

As the current density increased (0.5 A g^−1^ to 1.0 A g^−1^), the discharge time decreased rapidly, and the curves became compressed. This behavior arises because, under high current density conditions, the ion transport rate cannot keep pace with the applied load, restricting charge storage to only the easily accessible surface sites. Deeper penetration into the polymer bulk was limited, resulting in a reduced overall capacitance. The pronounced capacitance decay with increasing current density indicates that the device has limited high-rate charge-storage capability, rather than exceptionally high-rate capability. Despite this decline, the triangular shape was retained across all current density ranges, indicating that AC-based device maintains good reversibility and electrochemical integrity under dynamic cycling. The calculated specific capacitance values are listed in Table 4.

**Table 4:** Specific capacitance of AC-based device calculated from GCD profiles at different current densities

Current density (A g^−1^)	Specific capacitance (F g^−1^)
1.0	0.08
0.9	0.12
0.8	0.13
0.7	0.23
0.6	0.32
0.5	0.35
0.4	0.82
0.3	1.57
0.2	3.09
0.1	12.72
0.09	19.01

At the lowest current density of 0.09 A g^−1^, AC-based device exhibits a maximum specific capacitance of 19.01 F g^−1^, which gradually decreases to 12.72 F g^−1^ at 0.1 A g^−1^. As the current density continues to increase, the capacitance values steadily drop, reaching 0.08 F g^−1^ at 1 A g^−1^. This strong dependence is consistent with the diffusion-limited nature of polymer electrolytes, in which ionic motion and reorganization require sufficient time to fully contribute to charge storage. At higher current densities, the rapid charge–discharge cycles prevent ions from accessing the bulk interior, leaving only a fraction of the available storage sites active.

#### 3.8.3. Stability study

The long-term electrochemical stability of AC-based device was investigated by repeated GCD cycling over 3000 cycles, as shown in Fig. 10(c). Capacitance retention and coulombic efficiency were monitored simultaneously to evaluate both the durability of the charge storage and reversibility of the electrolyte system.

AC-based device exhibited good stability during prolonged cycling. Even after 3000 continuous cycles, the material retained 92.00% of its initial capacitance, indicating only a minor loss in charge storage ability over extended operation. Such high retention confirms that the electrolyte maintained its structural integrity and ionic transport pathways during repetitive cycling without significant degradation. Similarly, the coulombic efficiency remained stable, reaching 94.10% at the end of 3000 cycles. This value demonstrates that nearly all the charge stored during charging was recovered during discharge, reflecting minimal parasitic reactions and irreversible losses within the electrolyte. The decline observed in both parameters over time can be attributed to gradual adjustments at the electrode–electrolyte interface or minor resistive buildup, both of which are common in polymer-based systems. However, AC-based device exhibited high cycling stability, indicating stable electrochemical performance during prolonged cycling.

### 3.9. Electrochemical analysis of LIG-based device

The electrochemical performance of the LIG-based device with CPN30 electrolyte was evaluated using CV, GCD, and cycling stability studies, as discussed in the following sections.

#### 3.9.1. CV analysis

Fig. 11(a) shows the CV curves of the electrode recorded at scan rates ranging from 1 mV s^−1^ to 100 mV s^−1^. The CV curves maintained a quasi-rectangular shape across all scan rates, indicating predominantly capacitive charge storage behavior and good electrochemical reversibility. As the scan rate increased, the current density response increased accordingly, while the overall quasi-rectangular profile was retained, indicating predominantly capacitive behavior and reasonable electrochemical reversibility.

**Fig. 11:**
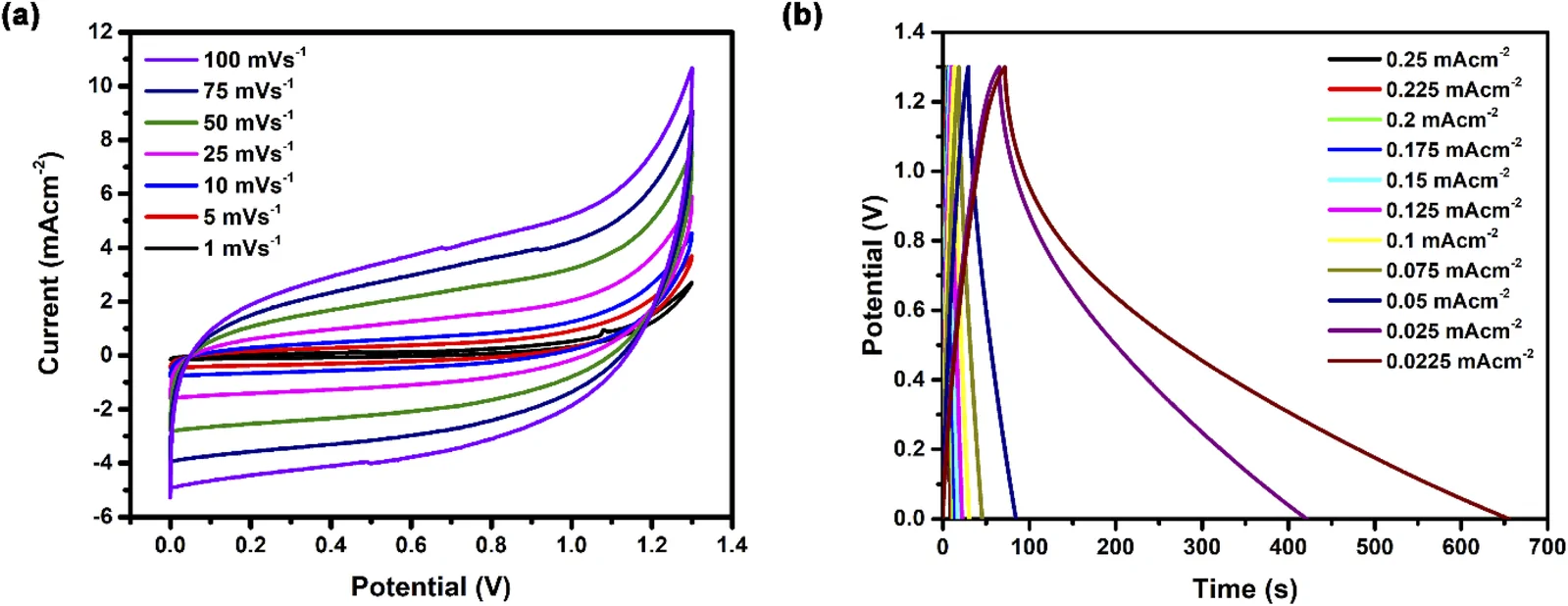
Electrochemical performance of the flexible LIG-based supercapacitor device: (a) CV curves recorded at different scan rates and (b) GCD profiles recorded at different current densities.

The areal capacitance values calculated from the CV curves are tabulated in Table 5, decreasing from 22.31 mF cm^−2^ at 1 mV s^−1^ to 8.26 mF cm^−2^ at 100 mV s^−1^. This decrease is attributed to the limited ion diffusion and electrolyte penetration into the deeper active sites at higher scan rates. At lower scan rates, the longer timescale allows ions to access a greater number of active sites and deeper pores, resulting in higher charge storage.

**Table 5:** Calculated areal capacitance of the device with LIG

Scan rate (mV s^−1^)	Areal capacitance (mF cm^−2^)
100	8.26
75	8.72
50	9.56
25	10.46
10	12.27
5	13.94
1	22.31

#### 3.9.2. GCD analysis

Fig. 11(b) shows the galvanostatic charge–discharge (GCD) curves of the device recorded at different applied current densities ranging from 0.0225 mA cm^−2^ to 0.25 mA cm^−2^. The GCD profiles exhibited capacitive charge–discharge behavior. The more pronounced difference between the charge and discharge times is mainly observed at the lower current densities, where the longer discharge duration reflects the greater time required for ion redistribution and release from the electrode–electrolyte interface. At higher current densities, the charge and discharge profiles become more compressed, and the time difference is less pronounced. This behavior is associated with the current-dependent ion transport and polarization of the device. As the current density increased, the discharge time decreased, which is characteristic of capacitive systems.

The areal capacitance values were calculated from the discharge curves and are summarized in Table 6. The capacitance decreased from 19.94 mF cm^−2^ at 0.0225 mA cm^−2^ to 1.70 mF cm^−2^ at 0.25 mA cm^−2^. The higher capacitance obtained at lower current density is attributed to sufficient time for electrolyte ions to access the active sites within the electrode structure. In contrast, at higher current density, only surface-accessible sites contribute to charge storage. The substantial decrease in areal capacitance with increasing current density indicates a pronounced rate-dependent capacitance loss. At higher current densities, the reduced time available for Na^+^ ion migration and access to electrochemically active sites limits effective charge storage.

**Table 6:** Areal capacitance of the LIG based device calculated from the GCD

Current density (mA cm^−2^)	Areal capacitance (mF cm^−2^)
0.25	1.70
0.225	1.87
0.2	1.93
0.175	2.04
0.15	2.20
0.125	2.38
0.1	2.67
0.075	3.19
0.05	4.29
0.025	13.77
0.0225	19.94

#### 3.9.3. Mechanical and cycling stability studies

The mechanical flexibility of the fabricated device was first demonstrated by physically bending the device into different configurations, as shown in Fig. 12. The device could be readily bent without visible damage or delamination, while maintaining electrical contact through the flexible current collectors. The corresponding electrochemical performance under different bending conditions was subsequently evaluated using CV and GCD measurements, as shown in Fig. 13.

**Fig. 12:**
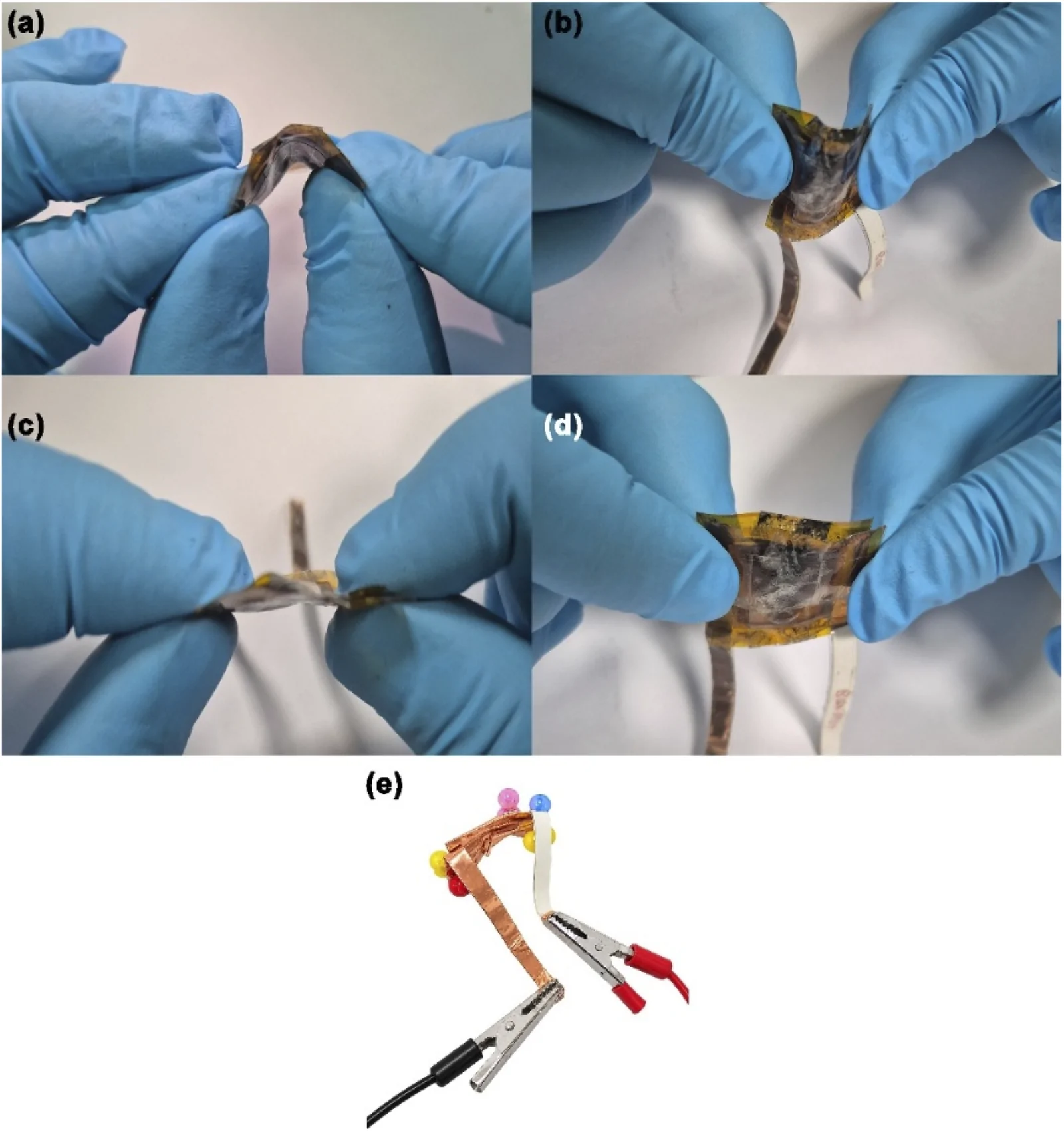
Photographs of the flexible device configurations and electrical connections used for electrochemical measurements: (a) flat configuration (180°), (b) 90° bent configuration, (c) side view of the flat configuration, (d) side view of the 90° bent configuration used for GCD measurements during bending-cycle tests, and (e) electrical connection used for CV measurements under bending conditions.

**Fig. 13:**
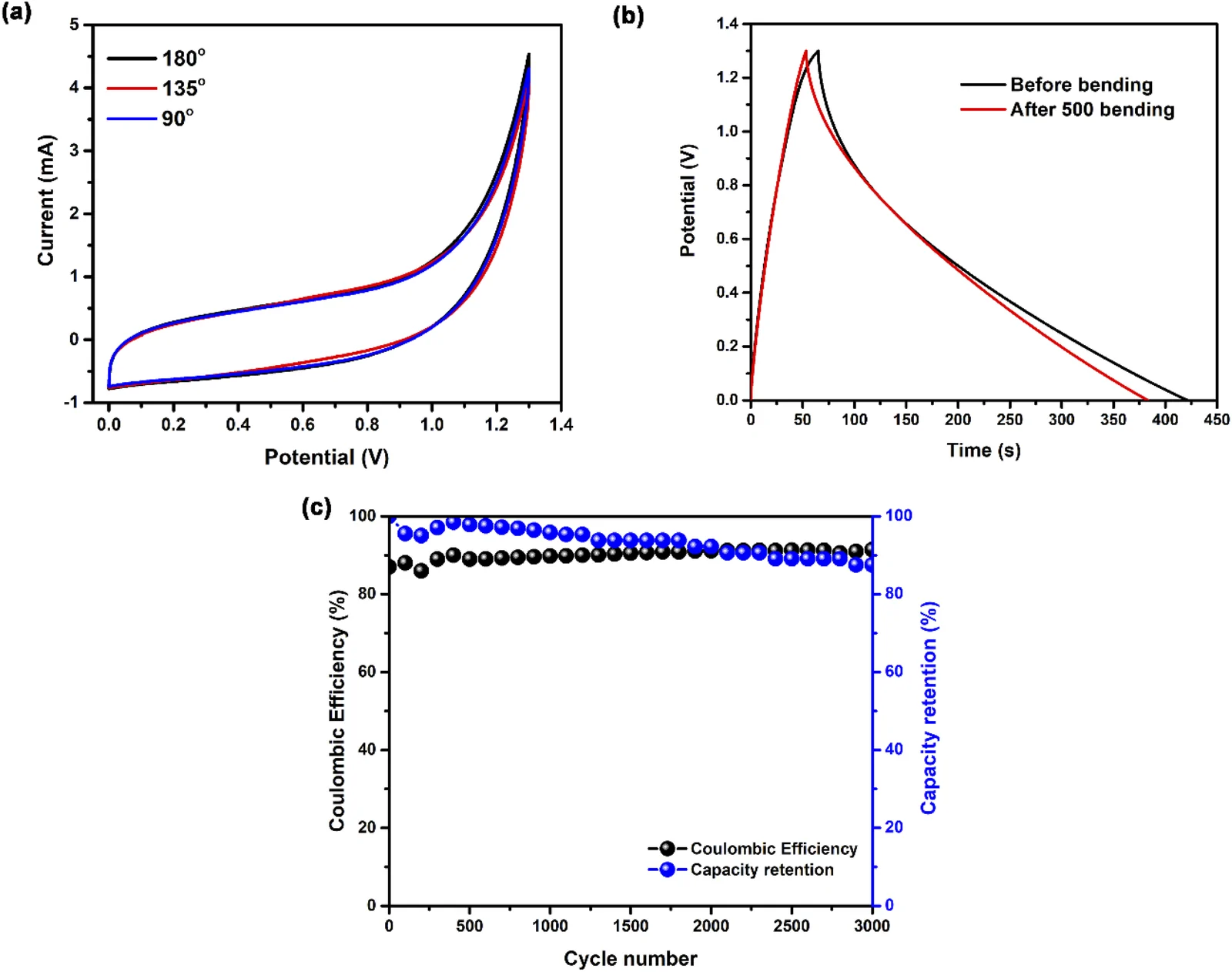
Mechanical flexibility and electrochemical stability of the LIG-based device: (a) CV curves recorded at different bending angles (b) GCD profiles recorded before and after 500 bending cycles (c) cycling performance showing capacitance retention and coulombic efficiency over 3000 cycles.

The flexibility of the device was evaluated by recording the electrochemical performance under different bending angles (90°, 135°, and 180°) (Fig. 13(a)). The CV curves exhibited similar shapes under all bending conditions, with only a slight change in the current response, indicating that the device maintains its capacitive behavior even under mechanical deformation. The calculated areal capacitance decreased marginally from 12.27 mF cm^−2^ at 180° to 11.65 mF cm^−2^ at 90°, corresponding to a capacitance retention of approximately 95.00%, confirming good mechanical flexibility of the device.

The mechanical durability of the device was further evaluated by performing repeated bending tests for up to 500 cycles. The GCD curves recorded before and after 500 bending cycles are shown in Fig. 13(b). The device retained 92.56% of its initial capacitance after 500 bending cycles, demonstrating good mechanical stability and flexibility. This decrease in performance after prolonged bending can be attributed to changes in interfacial contact or internal resistance; however, the overall electrochemical behavior remained largely preserved. These results clearly indicate that the device exhibits good flexibility and mechanical stability, making it suitable for flexible and wearable energy-storage applications.

The cycling stability of the device was evaluated by continuous charge–discharge measurements over 3000 cycles. As shown in Fig. 13(c), a gradual decrease in capacity during prolonged cycling is observed. After 3000 cycles, the device retained 87.52% of its initial capacity, indicating decent long-term stability. The coulombic efficiency of 91.42% at the end of 3000 cycles reflects reasonably reversible charge–discharge behavior and efficient charge transfer during repeated operation. The observed trends demonstrate retention of electrochemical function over the tested 3000-cycle laboratory protocol.

Tables 7 and 8 summarize the electrochemical performances of devices fabricated with the optimized CPN30 electrolyte and AC and LIG electrodes, respectively. The AC-based device exhibited good capacitive behavior and cycling stability, whereas the flexible LIG device demonstrated promising areal performance along with better mechanical flexibility and long-term stability, confirming the suitability of the developed electrolyte for both rigid and flexible solid-state supercapacitor applications.

**Table 7:** Electrochemical performance parameters of the device with AC

Parameter	Performance value
Specific capacitance at a scan rate of 1 mV s^−1^ obtained from CV (F g^−1^)	19.65
Specific capacitance at a current density of 0.09 A g^−1^ obtained from GCD (F g^−1^)	19.01
Energy density (W h kg^−1^)	4.46
Power density (W kg^−1^)	121.64
Capacitance retention (%) at the end of 3000 cycles	92.00
Coulombic efficiency (%) at the end of 3000 cycles	94.10

**Table 8:** Electrochemical performance parameters of the device with LIG

Parameter	Performance value
Areal capacitance at a scan rate of 1 mV s^−1^ obtained from CV (mF cm^−2^)	22.31
Areal capacitance at a current density of 0.0225 mA cm^−2^ obtained from GCD (mF cm^−2^)	19.94
Areal energy density (µW h cm^−2^)	4.68
Areal power density (mW cm^−2^)	0.029
Capacitance retention (%) during bending from 180° to 90°	95.00
Capacitance retention (%) after 500 bending cycles	92.56
Capacitance retention (%) after 3000 charge–discharge cycles	87.52
Coulombic efficiency (%) after 3000 charge–discharge cycles	91.42

### 3.10. Charge storage mechanisms of the fabricated devices

The ion transport mechanism in the CPN30 electrolyte is governed by the coordination of Na^+^ ions with the oxygen atoms of PEG and the hydroxyl and amine groups of CS. These interactions facilitate repeated association–dissociation processes, enabling Na^+^ ions to migrate through the polymer matrix. Simultaneously, ClO_4_^−^ ions interact with the polymer network through electrostatic and hydrogen bonding interactions. The increased amorphous character of the electrolyte enhances the polymer segmental motion and provides continuous pathways for ion hopping, resulting in a high IC and efficient charge transport.

The electrochemical performances of both the AC rigid device and LIG flexible device mainly arises from the electric double-layer capacitance (EDLC), which is based on reversible ion adsorption at the electrode–electrolyte interface.^45,46^ In the AC device, the high surface area and porous structure of the AC provide many active sites for ion accumulation. When a voltage is applied, Na^+^ and ClO_4_^−^ ions from the CPN30 solid-polymer electrolyte migrate and form an electrostatic charge layer at the electrode surface.^47^ The amorphous nature of the CS:PEG electrolyte facilitates ion movement by increasing polymer chain mobility, thereby supporting efficient ionic transport and stable charge storage.^48^ The rigid structure also maintains good contact between the electrode and the electrolyte, resulting in stable performance and good cycling behavior.

In the case of the LIG-based flexible device, the performance is supported by LIG's unique porous and interconnected structure. This structure provides continuous pathways for electron transport and open channels for ion diffusion. As a result, the device exhibits capacitive charge–discharge behavior, while ion diffusion and accessibility influence its rate-dependent performance. In addition, the flexibility of the LIG electrode allows it to withstand bending without breaking conductive pathways. Therefore, even under mechanical deformation, the electrochemical performance remains stable, confirming that the charge storage mechanism is preserved.

To further investigate the charge storage kinetics of the fabricated devices, Dunn's method was employed using scan rate-dependent CV measurements in a two-electrode configuration. The analysis was performed using the power-law relationship between the current response and scan rate, as expressed in eqn (12).^49^

12 *i* = *av*^*b*^

where *i* is the current at a selected voltage, *a* is the proportionality constant, *v* is the scan rate (mV s^−1^), and *b* is the kinetic parameter indicating the charge-storage mechanism.

Taking the logarithm of eqn (12) gives eqn (13):

13 log(*i*) = log(*a*) + *b* log(*v*)

The capacitive and diffusion-controlled charge-storage contributions were further quantified using Dunn's method according to eqn (14)

14 *i*(*V*) = *k*_1_*v* + *k*_2_*v*^1/2^

where *i*(*V*) is the current at a fixed potential, *v* is the scan rate, *k*_1_ represents the surface-controlled capacitive contribution, and *k*_2_*v*^1/2^ corresponds to the diffusion-controlled contribution of the electrode material.

As shown in Fig. 14(a), the *b*-values obtained for the AC- and LIG-based devices were 0.838 (*R*^2^ = 0.99) and 0.739 (*R*^2^ = 0.99), respectively. These values indicate mixed charge-storage kinetics with a substantial surface-controlled contribution in both devices. The relatively higher *b*-value of the AC-based device indicates a greater surface-controlled contribution compared with the LIG-based device, while the lower *b*-value of the LIG-based device indicates a comparatively greater contribution from diffusion-controlled processes. As shown in Fig. 14(b), the capacitive contribution of the AC-based device increased from 35.72% at 1 mV s^−1^ to 84.75% at 100 mV s^−1^, while the diffusion-controlled contribution decreased from 64.28% to 15.25%, respectively. This indicates that the contribution from surface-controlled charge storage becomes increasingly significant at higher scan rates.

**Fig. 14:**
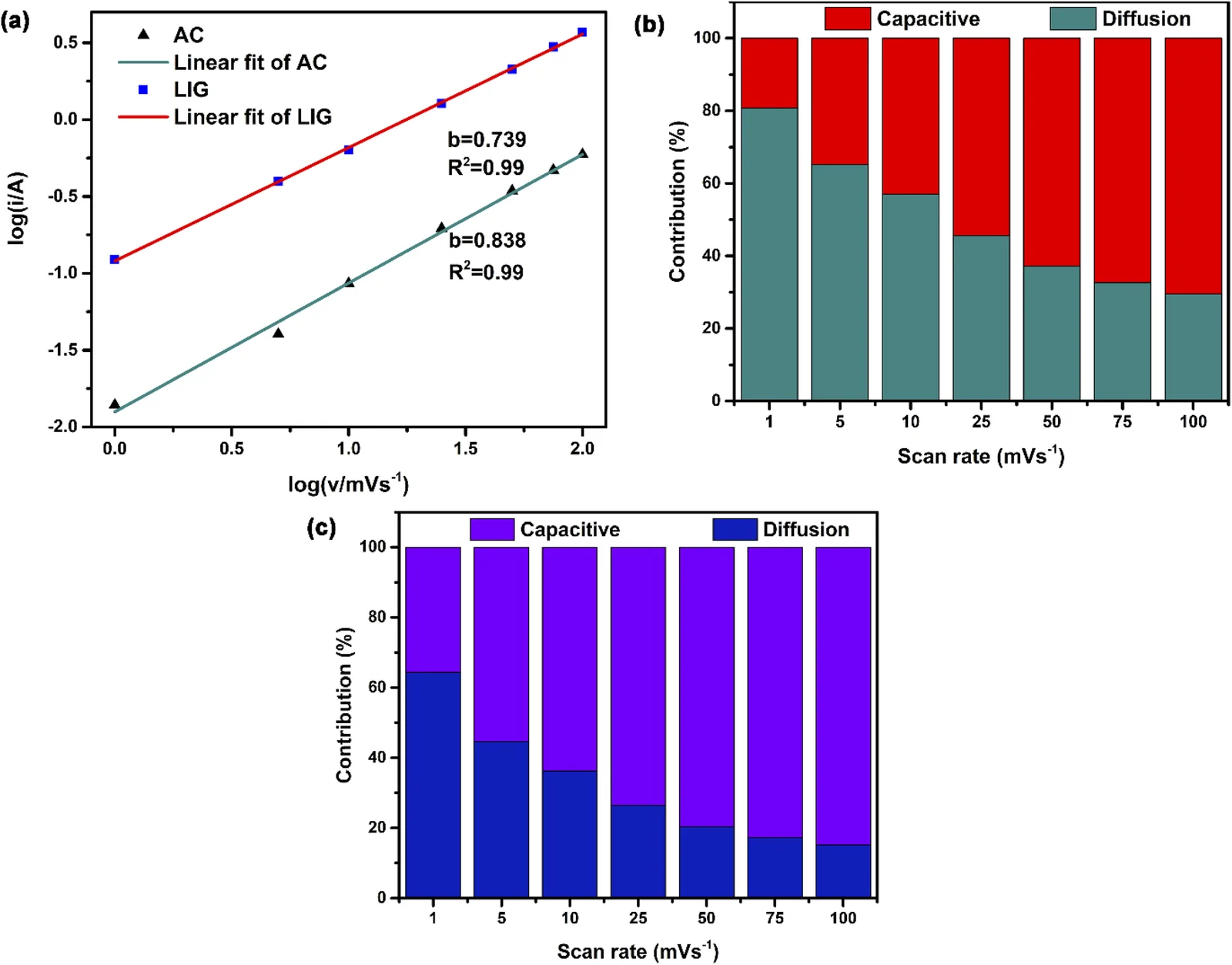
(a) *b*-Value analysis of the AC- and LIG-based supercapacitors, (b) capacitive and diffusion-controlled contributions for the AC-based device and (c) capacitive and diffusion-controlled contributions for the LIG based device.

For the LIG-based device, as shown in Fig. 14(c), the capacitive contribution increased from 19.25% at 1 mV s^−1^ to 70.44% at 100 mV s^−1^, whereas the diffusion-controlled contribution decreased from 80.75% to 29.56%. At lower scan rates, electrolyte ions have sufficient time to penetrate the porous electrode structure and access internal active sites, resulting in a greater diffusion-controlled contribution. As the scan rate increases, ion diffusion into deeper pores becomes kinetically limited, and charge storage increasingly occurs at readily accessible surface sites. Consequently, the capacitive contribution increases with increasing scan rate, whereas the diffusion-controlled contribution decreases. This behaviour is consistent with the porous structure of the carbon-based electrodes.

Building on our earlier CS:PEG–LiClO_4_ platform, the present work extends the same polymer host to NaClO_4_ and systematically investigates the influence of sodium-salt loading on the structure, ion transport, and electrochemical performance of the resulting biopolymer electrolyte. The main novelty lies in demonstrating the applicability of the optimized NaClO_4_-doped CS:PEG electrolyte in both rigid AC based and mechanically flexible LIG-based solid-state supercapacitors within the same study. Unlike studies that focus solely on electrolyte or electrode development, this work examines the relationship between electrolyte composition, polymer structure, ion transport, and device performance across different device configurations. Furthermore, the integration of the optimized biopolymer electrolyte with LIG electrodes provides a simple and potentially scalable approach for developing mechanically flexible all-solid-state energy-storage devices.

### 3.11. Electrochemical impedance spectroscopy analysis

EIS was employed to investigate the internal resistance and ion-transport characteristics of the fabricated supercapacitor devices. Fig. 15 shows the Nyquist plots and equivalent circuit fitting of the AC-based and LIG-based devices. The impedance spectrum was fitted using equivalent circuit models consisting of a series resistance (*R*_s_) connected to a parallel combination of charge-transfer resistance (*R*_ct_) and a constant-phase element (CPE, (*Q*)), as illustrated in Fig. 15(c) and (d). The CPE was incorporated to account for the non-ideal capacitive behavior associated with the porous electrode/electrolyte interface.

**Fig. 15:**
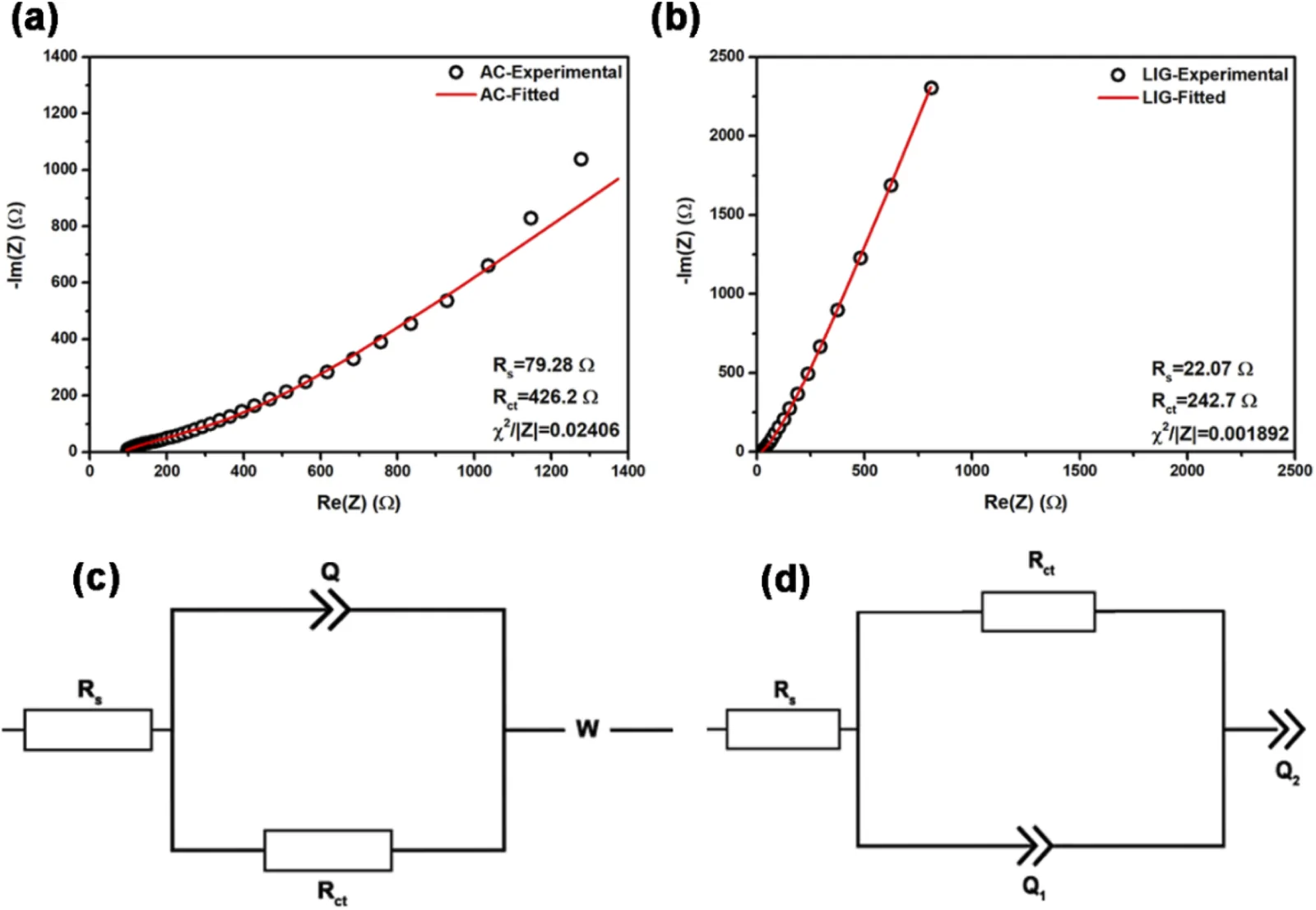
Nyquist plots of the (a) AC-based and (b) LIG-based devices, showing the experimental data and corresponding fitted curves. Equivalent electrical circuit models used for fitting the EIS data: (c) AC-based device and (d) LIG-based device.

The AC-based device exhibited a relatively high *R*_s_ of approximately 79.28 Ω, indicating higher internal resistance within the device. The corresponding *R*_ct_ was approximately 426.2 Ω, indicating comparatively higher resistance to charge transfer at the electrode/electrolyte interface. In comparison, the LIG-based device exhibited a substantially lower *R*_s_ of approximately 22.07 Ω, suggesting reduced internal resistance and improved electrical conductivity. The LIG-based device also exhibited a lower *R*_ct_ of approximately 242.7 Ω, indicating more facile charge transfer at the electrode/electrolyte interface. The lower *R*_s_ and *R*_ct_ values of the LIG-based device can be attributed to the interconnected porous graphene structure, which provides efficient electronic pathways and facilitates electrolyte-ion accessibility to the electrochemically active surface.

The equivalent-circuit fitting showed good agreement between the experimental and fitted impedance spectra. The corresponding *χ*^2^/|*Z*| values were 0.02406 and 0.001892 for the AC-based and LIG-based devices, respectively, indicating satisfactory fitting of the selected equivalent-circuit models to the experimental impedance data. The lower fitting error obtained for the LIG-based device further demonstrates good agreement between the experimental impedance response and the proposed equivalent circuit.

In addition, the Nyquist plot of the LIG-based device exhibited a nearly vertical low-frequency response, characteristics of predominantly capacitive behavior and favorable ion-transport characteristics. The lower *R*_s_ and *R*_ct_ together with the more vertical low-frequency response indicate favorable interfacial charge-transfer and ion-transport characteristics for the LIG-based device. These observations are consistent with the mixed charge-storage kinetics obtained from Dunn's analysis, where the LIG-based device exhibited a *b*-value of 0.739 and a substantial capacitive contribution. Overall, the EIS results demonstrate that the LIG-based device possesses lower internal and charge-transfer resistances and more favorable charge-transport characteristics than the AC-based device.

Table 9 compares the *σ* and electrochemical performance of the present CS:PEG:NaClO_4_ electrolyte with previously reported CS-based polymer electrolytes. Although some systems exhibit higher *σ* or capacitance values, these performances are often associated with the use of plasticizers, such as glycerol. The present electrolyte achieved a relatively high *σ* of 259.55 ± 19.88 µS cm^−1^ without the incorporation of glycerol while maintaining good cycling stability (92% capacitance retention after 3000 cycles). In addition, the electrolyte exhibited a high ionic transference number (0.9988), indicating that charge transport was predominantly ionic in nature. These results demonstrate that the developed CPN30 electrolyte provides a favorable balance between *σ*, electrochemical stability, and device performance for all-solid-state supercapacitor applications.

**Table 9:** Comparison of *σ* and electrochemical performance of chitosan-based polymer electrolytes reported for solid-state supercapacitors

Electrolyte	*σ* (µS cm^−1^)	Capacitance (F g^−1^)	Capacitance retention (%)	Ref.
CS:starch:glycerol:LiClO_4_	370	133.00 @ 10 mV s^−1^	—	50
CS:oxalic acid	0.495	—	—	51
CS:PVA:LiClO_4_	86.100	—	—	52
CS:dextran:NH_4_I:glycerol	344	108.30	100 after 100 cycles	53
CS:PEG:LiClO_4_	110	47.00 @ 10 mV s^−1^	94 after 1000 cycles	30
CS:PEG:LiClO_4_	127	5.32 @ 0.01 A g^−1^	84 after 3000 cycles	21
CS:PEG:NaClO_4_	259.55 ± 19.88	19.01 @ 0.09 A g^−1^	92 after 3000 cycles	This work

Table 10 compares the electrochemical performances of the present LIG-based supercapacitor and those of previously reported LIG-based all-solid-state supercapacitors. The developed device delivers an areal capacitance of 19.94 mF cm^−2^ at a current density of 0.0225 mA cm^−2^, which is higher than that of several reported LIG-based systems employing different polymer electrolytes. Although some reported devices exhibit superior long-term cycling retention, the present device combines a high areal capacitance with good cycling stability (87.52% retention after 3000 cycles). The enhanced performance can be attributed to the effective interaction between the LIG electrode and the optimized CPN30 electrolyte, which facilitates efficient transport and charge storage. These findings highlight the suitability of the developed electrolyte for flexible LIG-based all-solid-state energy storage devices.

**Table 10:** Comparison of the electrochemical performance of LIG-based all-solid-state supercapacitors reported in the literature

Electrode	Electrolyte	Capacitance (mF cm^−2^)	Capacitance retention (%)	Ref.
LIG	CS:PEG:LiClO_4_	18.83 @ 0.02 mA cm^−2^	89.3 after 3000 cycles	21
LIG	PVA/[EMIM][EtSO_4_]	15.10 @ 2 mA cm^−2^	80 after 3000 cycles	22
LIG	PVA/H_2_SO_4_	9.00 @ 0.02 mA cm^−2^	98 after 8000 cycles	54
LIG	PVA/H_2_SO_4_	13.27 @ 0.05 mA cm^−2^	103 after 1000 cycles	55
LIG	PPC/[EMIM][OTf]	1.75 @ 5 mV s^−1^	98.12 after 10 000 cycles	56
LIG	CS:PEG:NaClO_4_	19.94 @ 0.0225 mA cm^−2^	87.52 after 3000 cycles	This work

## 4. Conclusion

In this study, NaClO_4_-doped CS:PEG SPEs were successfully developed and optimized for all-solid-state supercapacitor applications. Structural analyses confirmed a strong interaction between the salt and polymer matrix, reducing crystallinity and increasing the amorphous phase, thereby facilitating ion transport. Thermal analysis revealed composition-dependent thermal behavior, with the electrolyte system exhibiting reasonable stability within the studied temperature range. Among all compositions, CPN30 exhibited the optimal *σ* (259.55 ± 19.88 µS cm^−1^), a wide ESW of 2.6 V, and an ionic transference number of 0.9988, confirming dominant ionic conduction behavior.

The optimized electrolyte was successfully integrated into both AC and flexible LIG-based symmetric devices. The AC-based device delivered a specific capacitance of 19.01 F g^−1^ at a low current density. The flexible LIG-based device achieved an areal capacitance of 22.31 mF cm^−2^ and exhibited good mechanical stability, capacitance retention found to be 92.56% after 500 bending cycles and 87.52% after 3000 charge–discharge cycles. These results indicate that the developed biopolymer electrolyte is suitable for flexible solid-state energy storage systems. The successful operation of the same optimized electrolyte in both rigid and flexible device architecture highlights its versatility for solid-state energy storage applications.

Overall, the results suggest that NaClO_4_-doped CS:PEG electrolytes are promising candidates for sustainable, flexible, solid-state supercapacitors. Further improvements may be achieved by modifying both the electrolyte and LIG electrodes. Adding nanofillers or plasticizers to the electrolyte may improve the *σ* and stability. Similarly, adding functional materials such as metal oxides, conducting polymers, or carbon nanostructures to the LIG electrode may enhance its conductivity and charge storage performance. These modifications may further improve the performance of flexible and wearable energy-storage devices.

## Conflicts of interest

The authors declare that they have no competing financial interests or personal relationships that could have influenced the work reported in this paper.

## Supplementary Material

RA-OLF-D6RA07397B-s001

## Data Availability

The data supporting this article has been included as part of the supplementary information (SI). Supplementary information: the detailed compositions and weight ratios of the CS:PEG:NaClO_4_ SPEs, glass-transition temperatures, equivalent-circuit fitting parameters, DSC thermograms, and CV optimization curves recorded at different voltage windows. See DOI: https://doi.org/10.1039/d6ra07397b.
