# Supplementary material for: Chitosan-poly(ethylene glycol)-NaClO4 solid polymer electrolytes for activated carbon and flexible laser-induced graphene-based all-solid-state supercapacitors
Source: RSC Adv. 2026 Oct 15;16(47):63014–34. doi: 10.1039/d6ra07397b (PMC13670042; doi:10.1039/d6ra07397b)
Supplement: Supplementary file 1 [file RA-016-D6RA07397B-s001.pdf]

Supplementary Information (SI) for RSC Advances.
This journal is © The Royal Society of Chemistry

aChitosan- poly (ethylene glycol)-$NaClO_4$ solid polymer electrolytes for activated carbon and flexible Laser-Induced Graphene-based all-solid-state supercapacitors

Jadan Resnik Jaleel UC[1], Somashekara Bhat[1*], Sudhakar YN[1], Vipin Cyriac[2]

[1]Manipal Institute of Technology, Manipal Academy of Higher Education, Manipal, India

[2] Symbiosis Institute of Technology (SIT), Pune Campus, Symbiosis International (Deemed University) (SIU), Pune-412115, India

[*]Corresponding author: soma.bhat@manipal.edu

Supplementary files

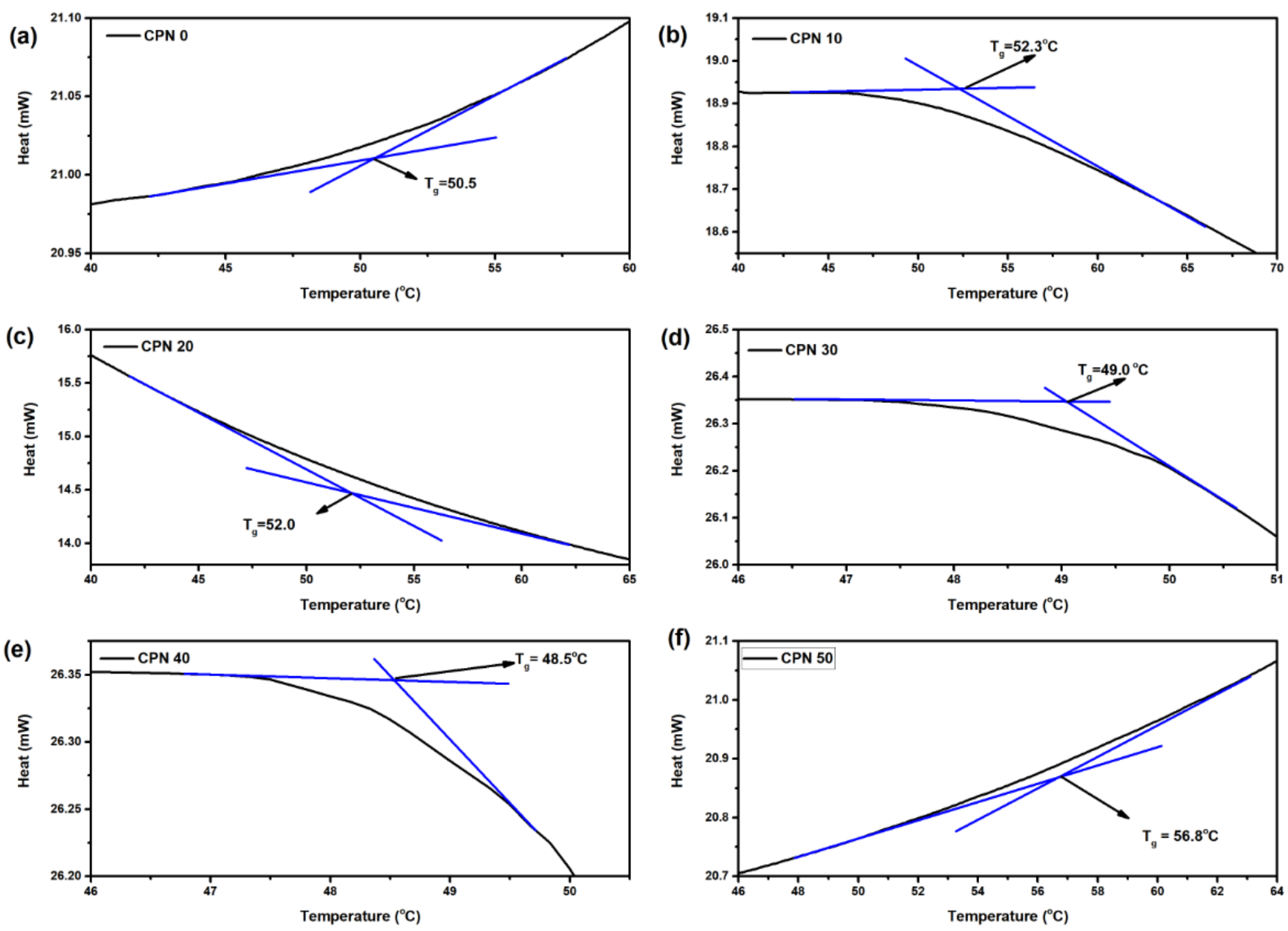

Figure S1. DSC thermograms of CS:PEG:$NaClO_4$ solid polymer electrolytes: (a) CPN0, (b) CPN10, (c) CPN20, (d) CPN30, (e) CPN40, and (f) CPN50.

Table S1. Composition and nomenclature of the CS:PEG:$NaClO_4$ solid polymer electrolytes.

Wt.% ratio of Chitosan: PEG	Amount of $NaClO_4$ (wt%)	Nomenclature
70:30	0	CPN0
	10	CPN10

	20	CPN20
	30	CPN30
	40	CPN40
	50	CPN50

Table S2. T_g values of CS-PEG-$NaClO_4$ electrolytes

Sample	T_g (°C)
CPN 0	50.5
CPN 10	52.3
CPN 20	52.0
CPN 30	49.0
CPN 40	48.5
CPN 50	56.8

Table S3. Equivalent-circuit fitting parameters obtained from the impedance spectra of CPN10-CPN50 solid polymer electrolytes

Sample	R_b (Ω)	Q1 (F s⁽$^{a-1}$⁾)	a1	χ²/|Z|
CPN 10	888 ± 25	0.274 × 10^{-6}	0.7389	1.281
CPN 20	267 ± 2.3	1.474 × 10^{-6}	0.6479	0.2263
CPN 30	41 ± 3.2	34.6 × 10^{-6}	0.4157	1.749 × 10⁻^{3}
CPN 40	69 ± 2.9	1.857 × 10^{-6}	0.7601	0.02117
CPN 50	228 ± 2.5	8.101 × 10^{-6}	0.5234	0.01927

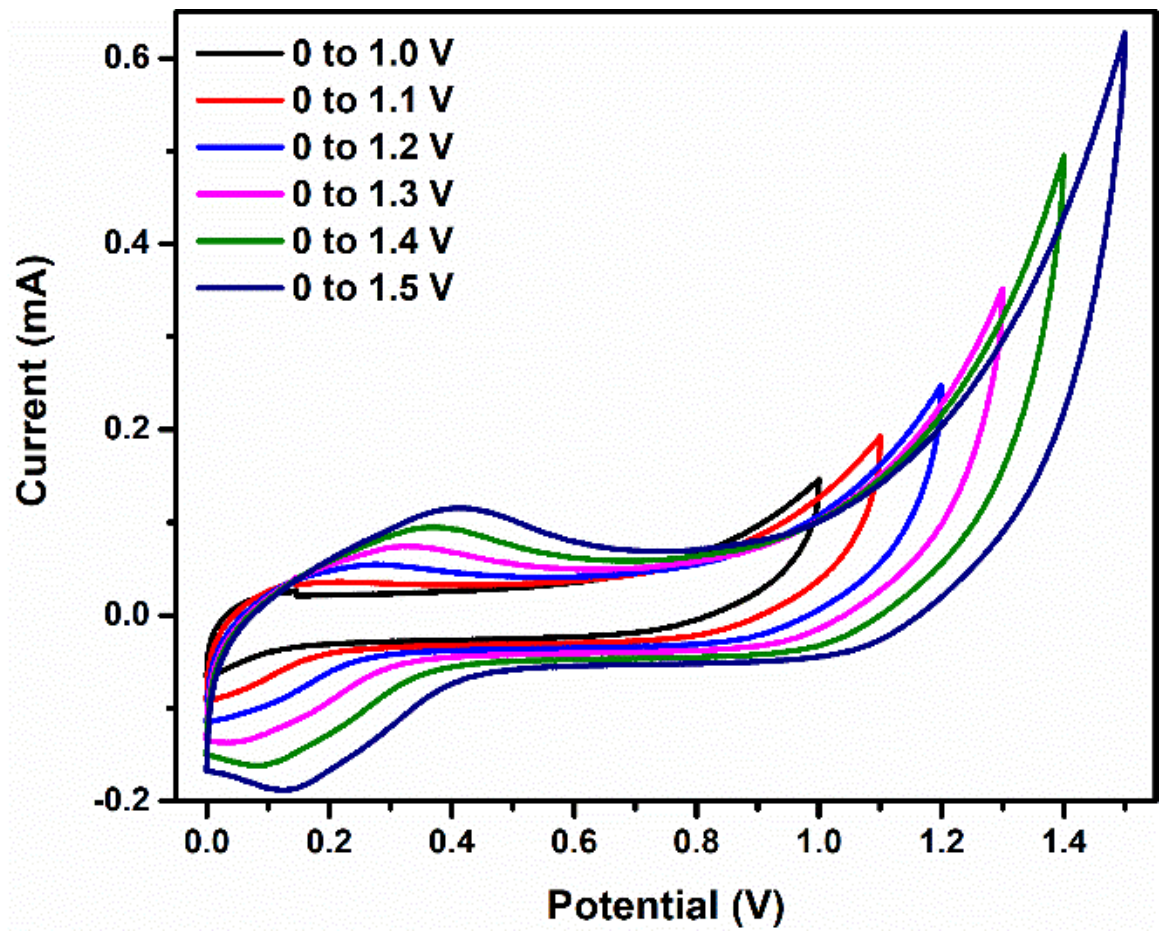

Figure S2. Operating voltage window optimization of the AC-based device with CPN30 electrolyte using CV curves recorded at a scan rate of 10 mV s^{-1} across voltage windows ranging from 0-1.0 to 0-1.5 V.
